# Down to the Last Dollar: Utilizing a Virtual Budgeting Exercise to Recognize Implicit Bias

**DOI:** 10.15766/mep_2374-8265.11199

**Published:** 2021-12-06

**Authors:** Christin Traba, Aditi Jain, Kimberly Pianucci, Jennifer Rosen-Valverde, Sophia Chen

**Affiliations:** 1 Assistant Professor, Department of Pediatrics, Rutgers New Jersey Medical School; 2 Second-Year Pediatric Resident, University of Pittsburgh Medical Center; 3 First-Year Pediatric Resident, Brown University; 4 Clinical Professor, Rutgers Law School

**Keywords:** Social Determinants of Health, Bias, Poverty, Food Insecurities, Case-Based Learning, Online/Distance Learning, Virtual Learning, Diversity, Inclusion, Health Equity

## Abstract

**Introduction:**

As social determinants of health and implicit bias are recognized as critical components of medical education, there is a need for novel approaches beyond didactics. We developed a small-group budgeting exercise to simulate the impact of poverty. Pediatrics exemplifies the effects of poverty on the family. This exercise allowed students to recognize the effects of food insecurities on health and reflect on biases regarding patients living in poverty.

**Methods:**

The virtual interactive budgeting exercise (1.5–2 hours) introduced third-year pediatric clerkship students to the challenges faced by a single parent living in poverty, requiring them to make choices on which budget items were most important. Students attempted to balance budgets within small breakout groups, followed by a group reflection on biases encountered. A faculty facilitator then debriefed with the larger group.

**Results:**

Within the first four rotations of the 2020–2021 academic year, 75 students completed the budgeting exercise and reflection, with 61 students completing the postexercise survey evaluation. Between 94% and 98% rated the objectives as met to a moderate, considerable, or very high degree. In addition, 98% of students noted the group discussion heightened their awareness regarding biases, and 95% agreed or strongly agreed the activity was conducted virtually without difficulty.

**Discussion:**

This simulated budgeting exercise provides a well-rounded experience for medical students, that can be administered at either the preclerkship or clerkship level, at a minimal cost, with interactive engagement of students in a virtual environment and reflection on biases within a group context.

## Educational Objectives

By the end of this activity, learners will be able to:

1. Illustrate the effects of social determinants, such as poverty and food insecurities, on health outcomes.
2. Reflect on biases regarding patients living in poverty and their health care adherence.

## Introduction

The pivotal role of social determinants of health (SDOH) in health care and medical education is well recognized. However, formalizing and implementing required SDOH curricula continue to evolve across the undergraduate to graduate medical education continuum. Per the World Health Organization, SDOH are the “conditions in which people are born, grow, live, work, and age.”^1^ Race, religion, socioeconomic status, food insecurity, and education are only a few of the many SDOH that precipitate differences in the health outcomes of patients. In 2008, the Commission on Social Determinants of Health noted the importance of recognizing and understanding health inequity in order to develop an action plan, as well as the need to invest in training of medical and health practitioners.^2^

Directly related to SDOH are adverse childhood experiences (ACEs), with studies demonstrating significantly higher ACEs exposure in individuals living in poverty, specifically those with income of less than $15,000 per year and those who were unemployed or unable to work.^3^ In 2016, the American Academy of Pediatrics published the policy statement “Poverty and Child Health in the United States” with recommendations for individual pediatricians to adapt the medical home, creating a team familiar with the effects and needs of families living in poverty.^4^ The core foundation of SDOH curricula must start in medical school.

At Rutgers New Jersey Medical School in Newark, New Jersey, we have a required longitudinal health equity and social justice course through the first 2 preclerkship years of medical school, which provides the foundation for SDOH, including curriculum on ACEs and bias. Health equity content is threaded throughout the third- and fourth-year required clerkships to reinforce key concepts and provide opportunities for reflection tied to daily clinical experiences. In pediatrics, we worked with our medical legal partnership at Rutgers Law School to develop and implement a curriculum.

SDOH curricula published in *MedEdPORTAL* over recent years span from first-year medical students to clerkship students and, ultimately, residents. Angela Y. Song and colleagues published an introduction to SDOH curriculum for first-year medical students. This curriculum is expansive, including access to care, food insecurity, human trafficking, immigrant health, LGBT health, race and ethnicity, and women's health.^5^ Within the food insecurity case, students complete a brief budgeting exercise where they budget one meal for a family of five within a 15-minute period. For third-year pediatric clerkship students, Dr. Melanie C. Marsh and colleagues published a didactic curriculum utilizing an interactive discussion of advocacy and SDOH specific to children.^6^

The Community Pediatrics Training Initiative of the American Academy of Pediatrics published a project planning tool in *MedEdPORTAL*, which focuses on pediatric residency trainees as an individual or group exercise with guided reflection.^7^ Furthermore, residency programs and medical schools have implemented the Missouri Community Action Network poverty simulation kit; the kits cost $2,300.^8^ This live simulation with role-play demonstrates positive outcomes in graduate medical education.^9^ However, implementation of the role-play during the COVID-19 pandemic can be problematic, and the role-play can be cost prohibitive.

As the social determinants affecting children's health are intertwined with their overall family environment, we sought to create an exercise in which students would gain perspective for the daily challenges parents face. We wanted to implement an exercise that provided a simulated environment that could be replicated within each pediatric clerkship, which meant administering the curriculum eight times per year (6-week clerkship). As a result, logistically, it would have been quite difficult to host a live simulation with role-play repeatedly throughout the year. We felt strongly that the session should be within the pediatric clerkship as opposed to the whole class in the transition to clerkship phase. We wanted students to have the clinical experience and immersion during pediatrics to provide perspective for the simulation.

Within the third-year pediatric clerkship, we implemented a zero-cost, small-group, simulated, virtual budgeting exercise to expose students to the effects of poverty and food insecurities on health as well as to provide them with the opportunity to reflect on individual biases regarding patients living in poverty and their health care adherence. In reviewing prior publications, we found only one that utilized a budgeting exercise; this asked the student to budget for one meal for a family of five within a 15-minute period and was part of a larger curriculum.^5^

Our approach is unique in that students complete the family budget for an entire month as opposed to just one meal, taking into account not just food but also housing, clothing, personal hygiene, and utilities within a virtual environment. In addition, students complete a group reflection exercise, facilitating further discussion on implicit biases. As prior studies have shown, medical students demonstrate an implicit preference for White persons and upper social class, as do the general nonmedical population.^10^ Our exercise allows students to reflect and recognize such biases as the major first step in mitigating their adverse effects while immersing themselves for a brief period in the parents' shoes.

## Methods

We implemented the small-group, virtual, simulated budgeting exercise in the 2020–2021 academic year within the third-year pediatric clerkship in the midst of the COVID-19 pandemic. All students were required to complete this session as part of their required clerkship didactics. The first four rotations of the academic year (June-November 2020) were included. The Rutgers Newark Health Sciences Institutional Review Board approved this study as exempt on August 21, 2020 (Pro2020002771).

### Background Resources and Preparation

Students had completed the longitudinal health equity and social justice course in the preclerkship years, including workshops on ACEs and addressing racism in health previously published in *MedEdPORTAL.*^11,12^

In the pediatric clerkship, we provided a 30-minute podcast reinforcing key concepts on SDOH, including key definitions in health equity, poverty, ACEs, and a framework for addressing implicit bias (Appendix A).

### Case Scenario (10 Minutes)

The case scenario provided to the students highlighted a 26-year-old single parent of two children, ages 10 years and 8 months old. The facilitator read the case to all students at the start of the session and then reviewed the outline of the session, tasking the students to create a monthly budget based on the designated income. Students were provided with the following documents in an Excel spreadsheet shared in a virtual platform:

• Case scenario (Appendix B).
• Budget form, including housing, utilities, telephone, food, cleaning products, personal care, transportation, and others (Appendix C, spreadsheet 1).
• Food budget form, including a sample of foods/items they could use (Appendix C, spreadsheet 2).
• List of common food prices, a comparison of grocery costs from a local food store, chain grocery store, and membership-only warehouse (Appendix C, spreadsheet 3). These prices were obtained online from the grocery stores in the local area at the time of publication.

Examples of a completed budget were provided (Appendices D and E).

### Breakout Groups (45 Minutes)

Students were randomly assigned into groups of three to four via breakout groups (the maximum total number of students within one rotation was 23) utilizing a virtual platform. Each group had an online, shared document with all of the above information, allowing each group to work independently and be able to view their own progress in real time. For example, if there were five groups of three students, each group had a separate budgeting form to complete that all members of the group could review via a shared online document. The facilitator checked in virtually with each group and answered questions as needed.

### Group Reflection Exercise (15 Minutes)

At 45 minutes, all groups completed the reflection assignment and submitted it to the online learning management system. Each group submitted one response to the question “What stereotypes or biases (positive and/or negative) did you come across during this budgeting exercise?” (Appendix B).

### Large-Group Debrief (30 Minutes)

After the group reflections were completed, all students returned to the larger group, where the facilitator debriefed with the students. Each group shared components of their group reflection. The facilitator reviewed several next steps or what-if questions. Examples included the following:

• What factors lead to a child with food insecurities becoming obese?
• What if the 8-month-old child is admitted for a prolonged hospitalization? In a single-parent household, how would they balance the care of both children?
• Discuss transportation issues for the single parent in shopping for groceries and/or attending doctors' visits.

A facilitator guide (Appendix F) was provided with examples of biases identified through the exercise as well as the what-if questions.

### Postexercise Survey

At the conclusion of the activity, students completed an anonymous postexercise survey regarding their own budgeting practices, the effectiveness of the exercise in meeting its objectives, and the ease of the virtual delivery (Appendix G).

## Results

A total of 75 students completed this curriculum within the 2020–2021 academic year between June and November 2020 (four of eight rotations within the academic year). Sixty-one students completed the postexercise survey (81% response rate).

Only 20% of students (*n* = 12) reported utilizing a personal budget when making regular purchases. The majority of students noted shopping for themselves (58%, *n* = 36), with an additional 18% (*n* = 11) noting someone else did the shopping. Twenty percent (*n* = 12) of students noted shopping for two to three persons and only 3% (*n* = 2) for four or more persons.

In general, the majority of students noted that the budgeting exercise met the learning objectives, ranging from 93% to 98% rating that the objectives had been met to a moderate, considerable, or very high degree (Table 1). In addition, 98% of students noted that the group discussion enhanced their perspective about biases towards patients to a moderate, considerable, or very high degree.

**Table 1.**
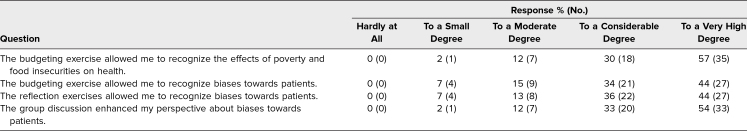
Students' Rating of the Budgeting Exercise's Effectiveness (*N* = 61)

Ninety-five percent of students agreed or strongly agreed the exercise was conducted virtually without difficulty, with 80% rating the time as sufficient. The vast majority of students (93%) also recognized the relationship of the budgeting exercise to patients they had seen during the pediatric clerkship (Table 2).

**Table 2.**
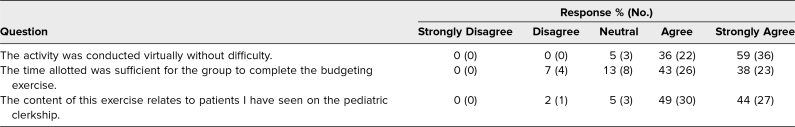
Students' Rating of the Budgeting Exercise's Implementation and Its Relationship to Their Pediatric Clerkship Experiences (*N* = 61)

In the narrative assessment of strengths of the curriculum, students overwhelmingly noted increased perspective and/or insight into patients' experiences (the term *eye-opening* was used more than once), with three major themes identified: the utility of experiential learning, reflecting on biases, and linking social determinants to health (Table 3). In the narrative assessment regarding areas for improvement, students noted more time needed for completion of the budgeting exercise. Within the first rotation, they also noted it would be helpful to have a separate spreadsheet for just the food, which was implemented in the subsequent second rotation. Several students also suggested the addition of different vignettes per group to see how different situations could affect budgeting.

**Table 3.**
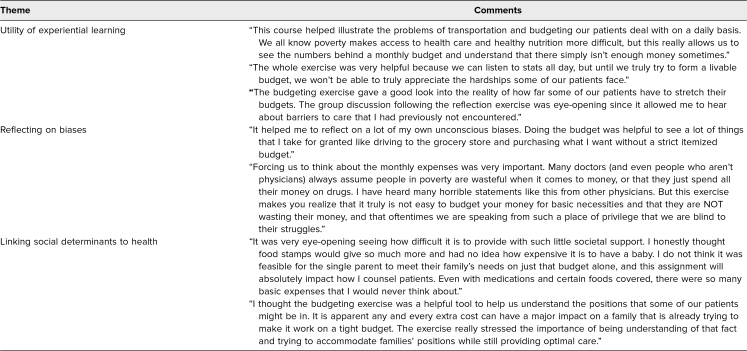
Students' Narrative Comments on the Budgeting Exercise

## Discussion

To address the need for an expanded health equity curriculum within the pediatric clerkship, we implemented a virtual, simulated budgeting exercise, providing an opportunity for students to reflect on biases related to food insecurity, budgeting, and poverty. Student evaluation of the exercise noted the session was successful in meeting the objectives in identifying the effects of poverty on health as well as the ability to reflect on biases towards patients and families in a small-group setting. Students also rated the virtual nature of the exercise highly, highlighting that a zero-cost simulated exercise can result in meaningful reflections. We found that only a small percentage of students currently used a budget when making regular purchases and that most students did not shop for more than one person. Narrative responses showed that the exercise enabled students to identify specific concerns that they had not previously considered, including transportation costs, the limited support from food assistance programs, and the challenges that can arise in providing for young children.

The interactive nature allowed students to immerse themselves in the simulated budgeting exercise and to experience the extraordinary circumstances families face. They were then able to link the effects of poverty and social determinants to the health of the children and family overall. This provided a practical hands-on approach to augment the traditional didactic format of an SDOH curriculum. The group reflection fostered empathy while allowing reflection on both individual and societal biases. Implicit bias can affect clinical decision-making, which may further contribute to health inequities.^13^ The facilitated discussion allowed these links to be highlighted, utilizing the what-if questions.

For example, a bias frequently noted on the inpatient service is towards parents who are not at the bedside day and night with their child, signifying that they are not caring or good parents. The discussion concerned how a single parent would have to care for their other child while one was admitted to the hospital. Students put themselves in the shoes of this single parent and reflected on what they would do in this situation, quickly realizing they would have no choice but to stay home with the other child. A second example is the labeling of a family/patient as noncompliant with a medication. This budgeting exercise allowed students to immerse themselves in the role of a single parent living on a tight budget and revealed the lack of additional funds to pay for any medication not covered by insurance. While we can provide examples and stories to help students learn, an immersive, simulated experience can give a broader perspective.

A portion of students noted that the time was sufficient for the allotted exercise. Based on narrative comments, this reflected the 45-minute allotted time for the budgeting exercise itself. While not every group may be able to solve the budgeting exercise in the allotted time, we found that students overwhelmingly were still able to identify barriers and challenges. Since the point of the exercise was not to solve the budget but instead to identify barriers and challenges, we opted not to expand the time for the budgeting exercise.

With regard to limitations, we must be mindful during the case discussion to be sensitive to the voices of the students, reinforce basic concepts such as the single parent being an equal to the physician, and avoid reinforcing biases. Inevitably, students and the facilitator may make biased statements that need to be acknowledged. Depending on the situation, the facilitator may choose to meet with the student privately or, if appropriate, address the group if there is a general concept for students to learn. It is important to create a safe and guilt-free environment for the students to participate in while also mitigating bias. The facilitator needs to recognize their own bias as well and feel empowered to address it in front of the group. For example, the parent in the case was purposefully gender neutral. The facilitator referred to the parent as she or her, which led to discussion regarding gender bias and assuming the parental role to be a woman. The faculty facilitator also needs to have a background in SDOH, be familiar with related terms such as ACEs, immigration regulations, and insurance requirements, and be comfortable in hosting a group debrief on sensitive topics such as bias. At Rutgers New Jersey Medical School, we had the same faculty facilitator lead each session, which helped the ease of implementation, as the facilitator built the comfort level in debriefing on such sensitive topics. While this may be challenging depending on resources, we recommend having a core group of facilitators to debrief with each other intermittently in order to identify concerns or need for adaptations to the exercise. The case scenario can also be adapted to limit expanded topics. For example, immigration status can be changed to a United States citizen to remove a component of the discussion.

The evaluation was subjective in that students were rating the degree to which objectives had been met as well as the utility of the virtual environment. There was no formalized objective assessment to measure knowledge gained. Next steps to improve the exercise include the expansion of the survey to include a pre- and postexercise survey comparison to obtain more robust outcome measures. This may be particularly helpful in assessing recognition of implicit bias before and after the exercise.

Another challenge is generalizability across different geographic regions. While food costs and benefits may vary based on state or region, the overall purpose of this exercise is to immerse students for a brief time into what a family may face in juggling social, financial, and medical concerns. Thus, the food costs and/or state-specific benefits do not need to be altered based on geographic location if this is explicitly noted to the students. However, these can be easily adapted using the resources noted in the facilitator's guide (Appendix F). Learners can be encouraged to search for food prices online using grocery stores near the populations served at their medical school or hospital.

In summary, this simulated budgeting exercise encourages students to examine and understand how hard it is for parents to make agonizing decisions, recognizing that these choices are not personal but are forced by societal structures. The group reflection and debriefing can facilitate discussion regarding the racism and discrimination built into these societal structures. It is an exercise in empathy and understanding the realities of what children and families living in poverty face and the adverse effects of structural determinants, not personal behaviors and choices, on health. While this exercise was taught within a third-year pediatric clerkship, it could be used for varying levels of learners from undergraduate through graduate and even continuing medical education, as well as with different specialties, including in an interprofessional setting.

## Appendices


Social Determinants of Health Lecture.pptxCase Scenario with Group Reflection Exercise.docxBudgeting Templates - Common Food Prices.xlsxExample of Budget - Chain Grocery Store.xlsxExample of Budget - Wholesale Grocery Store.xlsxFacilitator Guide.docxSession Evaluation.docx
*All appendices are peer reviewed as integral parts of the Original Publication.*

## References

[1] Social determinants of health. World Health Organization. Accessed January 11, 2021. https://www.who.int/health-topics/social-determinants-of-health#tab=tab_1

[2] Commission on Social Determinants of Health. Closing the Gap in a Generation: Health Equity Through Action on the Social Determinants of Health. World Health Organization; 2008. Accessed January 11, 2021. https://www.who.int/social_determinants/final_report/csdh_finalreport_2008.pdf 10.1016/S0140-6736(08)61690-6 18994664

[3] Merrick MT, Ford DC, Ports KA, Guinn AS. Prevalence of adverse childhood experiences from the 2011–2014 Behavioral Risk Factor Surveillance System in 23 states. JAMA Pediatr. 2018;172(11):1038–1044. 10.1001/jamapediatrics.2018.2537 30242348 PMC6248156

[4] Council on Community Pediatrics. Poverty and child health in the United States. Pediatrics. 2016;137(4):e20160339. 10.1542/peds.2016-0339 26962238

[5] Song AY, Poythress EL, Bocchini CE, Kass JS. Reorienting orientation: introducing the social determinants of health to first-year medical students. MedEdPORTAL. 2018;14:10752. 10.15766/mep_2374-8265.10752 30800952 PMC6342337

[6] Marsh MC, Supples S, McLaurin-Jiang S, Brown CL, Linton JM. Introducing the concepts of advocacy and social determinants of health within the pediatric clerkship. MedEdPORTAL. 2019;15:10798. 10.15766/mep_2374-8265.10798 30800998 PMC6376941

[7] Hoffman BD, Rose J, Best D, et al. The Community Pediatrics Training Initiative project planning tool: a practical approach to community-based advocacy. MedEdPORTAL. 2017;13:10630. 10.15766/mep_2374-8265.10630 30800831 PMC6338167

[8] Poverty simulations. Missouri Community Action Network. Accessed January 11, 2021. https://www.communityaction.org/povertysimulations/

[9] Maguire MS, Kottenhahn R, Consiglio-Ward L, Smalls A, Dressler R. Using a poverty simulation in graduate medical education as a mechanism to introduce social determinants of health and cultural competency. J Grad Med Educ. 2017;9(3):386–387. 10.4300/JGME-D-16-00776.1 28638532 PMC5476403

[10] Haider AH, Sexton J, Sriram N, et al. Association of unconscious race and social class bias with vignette-based clinical assessments by medical students. JAMA. 2011;306(9):942–951. 10.1001/jama.2011.1248 21900134 PMC3684149

[11] DallaPiazza M, Padilla-Register M, Dwarakanath M, Obamedo E, Hill J, Soto-Greene ML. Exploring racism and health: an intensive interactive session for medical students. MedEdPORTAL. 2018;14:10783. 10.15766/mep_2374-8265.10783 30800983 PMC6354798

[12] Pletcher BA, O'Connor M, Swift-Taylor ME, DallaPiazza M. Adverse childhood experiences: a case-based workshop introducing medical students to trauma-informed care. MedEdPORTAL. 2019;15:10803. 10.15766/mep_2374-8265.10803 30931382 PMC6415008

[13] van Ryn M, Saha S. Exploring unconscious bias in disparities research and medical education. JAMA. 2011;306(9):995–996. 10.1001/jama.2011.1275 21900142 PMC4169280

