# Supplementary material for: Down to the Last Dollar: Utilizing a Virtual Budgeting Exercise to Recognize Implicit Bias
Source: MedEdPORTAL. 2021 Dec 6;17:11199. doi: 10.15766/mep_2374-8265.11199 (PMC8645532; doi:10.15766/mep_2374-8265.11199)
Supplement: Supplementary file 1 — Social Determinants of Health Lecture.pptxCase Scenario with Group Reflection Exercise.docxBudgeting Templates - Common Food Prices.xlsxExample of Budget - Chain Grocery Store.xlsxExample of Budget - Wholesale Grocery Store.xlsxFacilitator Guide.docxSession Evaluation.docx [file mep_2374-8265.11199-s001.zip › A. Social Determinants of Health Lecture.pptx]

## Slide 1
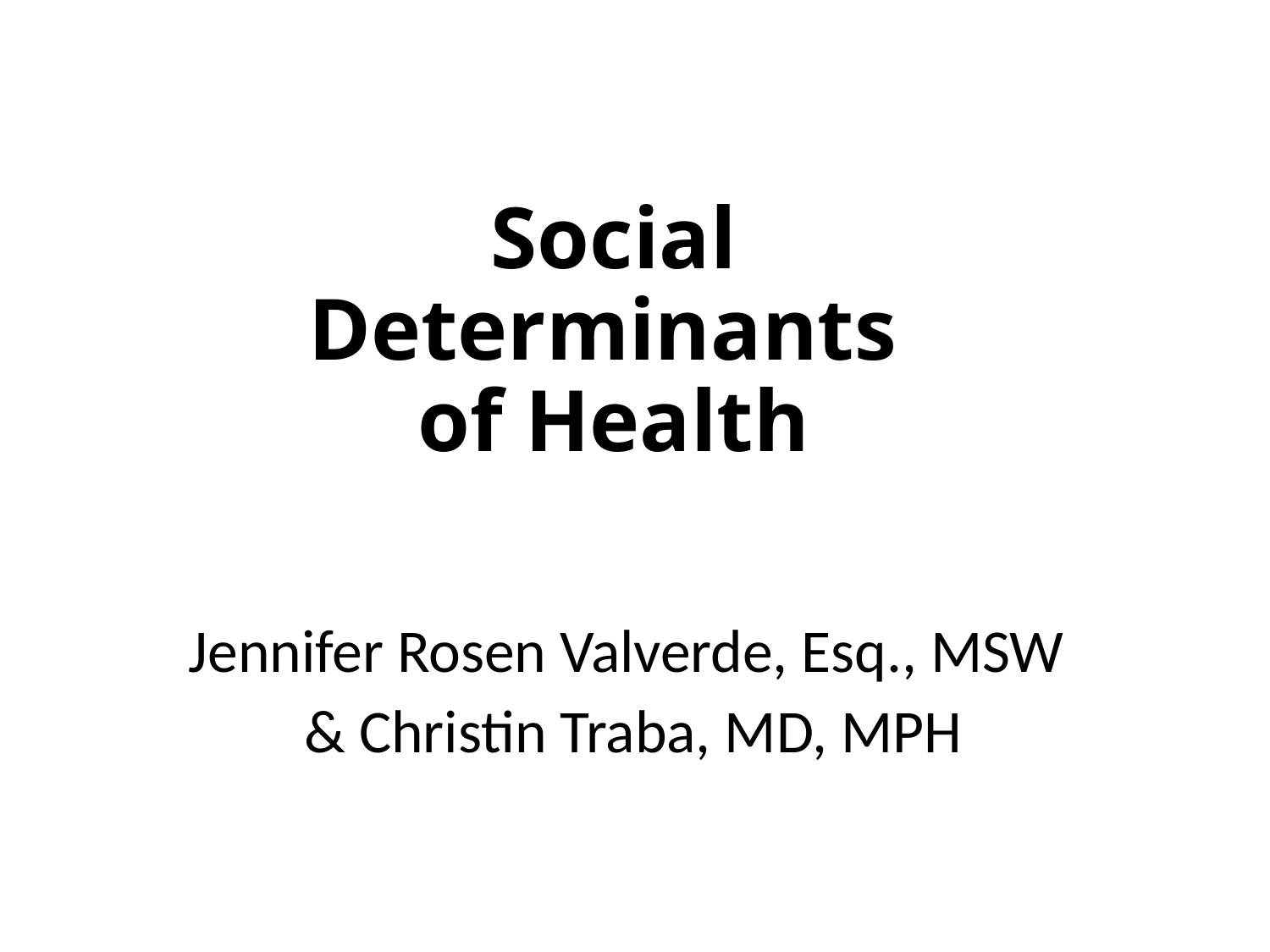

# Social Determinants of Health
Jennifer Rosen Valverde, Esq., MSW
& Christin Traba, MD, MPH

## Slide 2
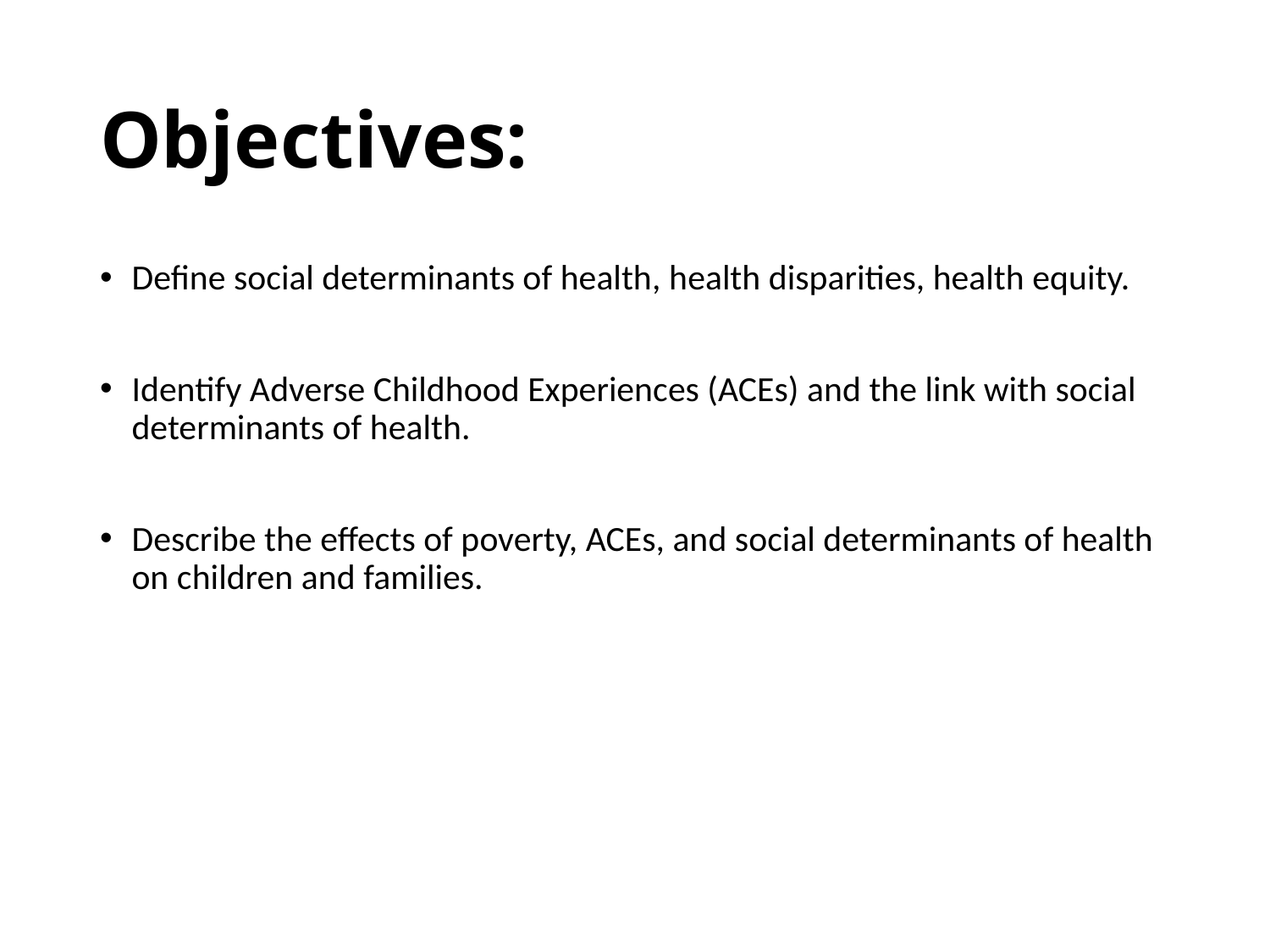

# Objectives:
Define social determinants of health, health disparities, health equity.
Identify Adverse Childhood Experiences (ACEs) and the link with social determinants of health.
Describe the effects of poverty, ACEs, and social determinants of health on children and families.

## Slide 3
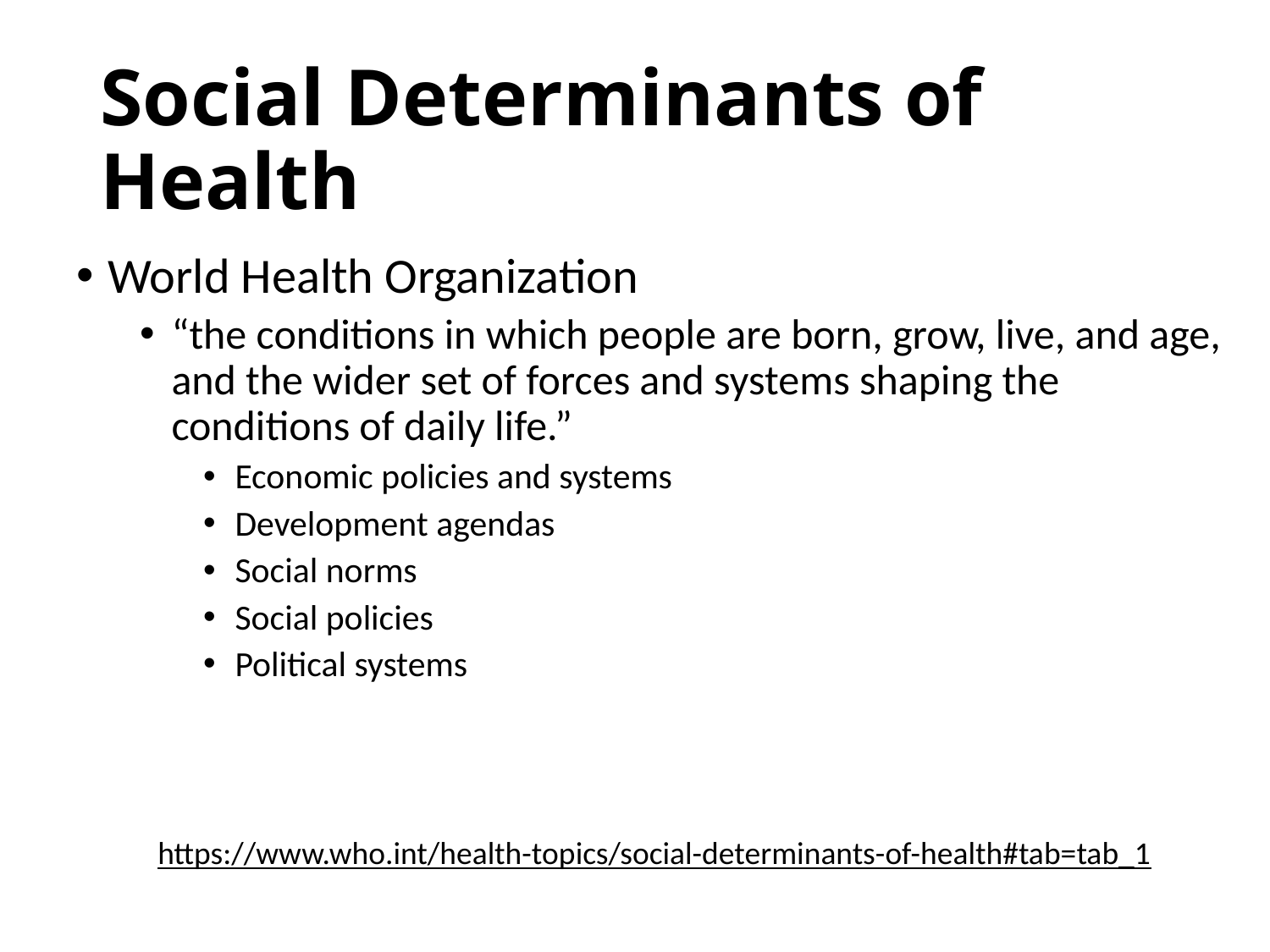

# Social Determinants of Health
World Health Organization
“the conditions in which people are born, grow, live, and age, and the wider set of forces and systems shaping the conditions of daily life.”
Economic policies and systems
Development agendas
Social norms
Social policies
Political systems
https://www.who.int/health-topics/social-determinants-of-health#tab=tab_1

## Slide 4
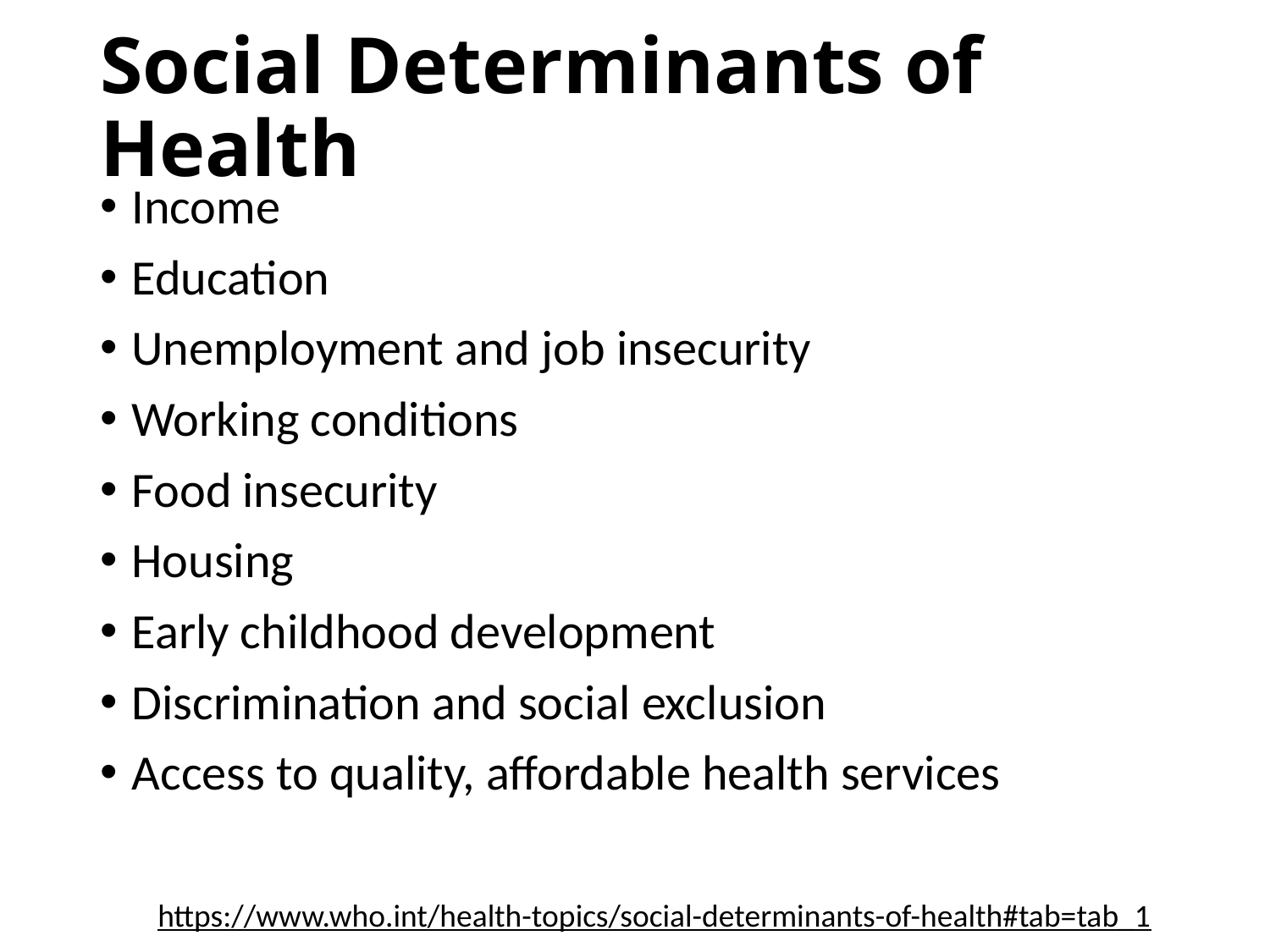

# Social Determinants of Health
Income
Education
Unemployment and job insecurity
Working conditions
Food insecurity
Housing
Early childhood development
Discrimination and social exclusion
Access to quality, affordable health services
https://www.who.int/health-topics/social-determinants-of-health#tab=tab_1

## Slide 5
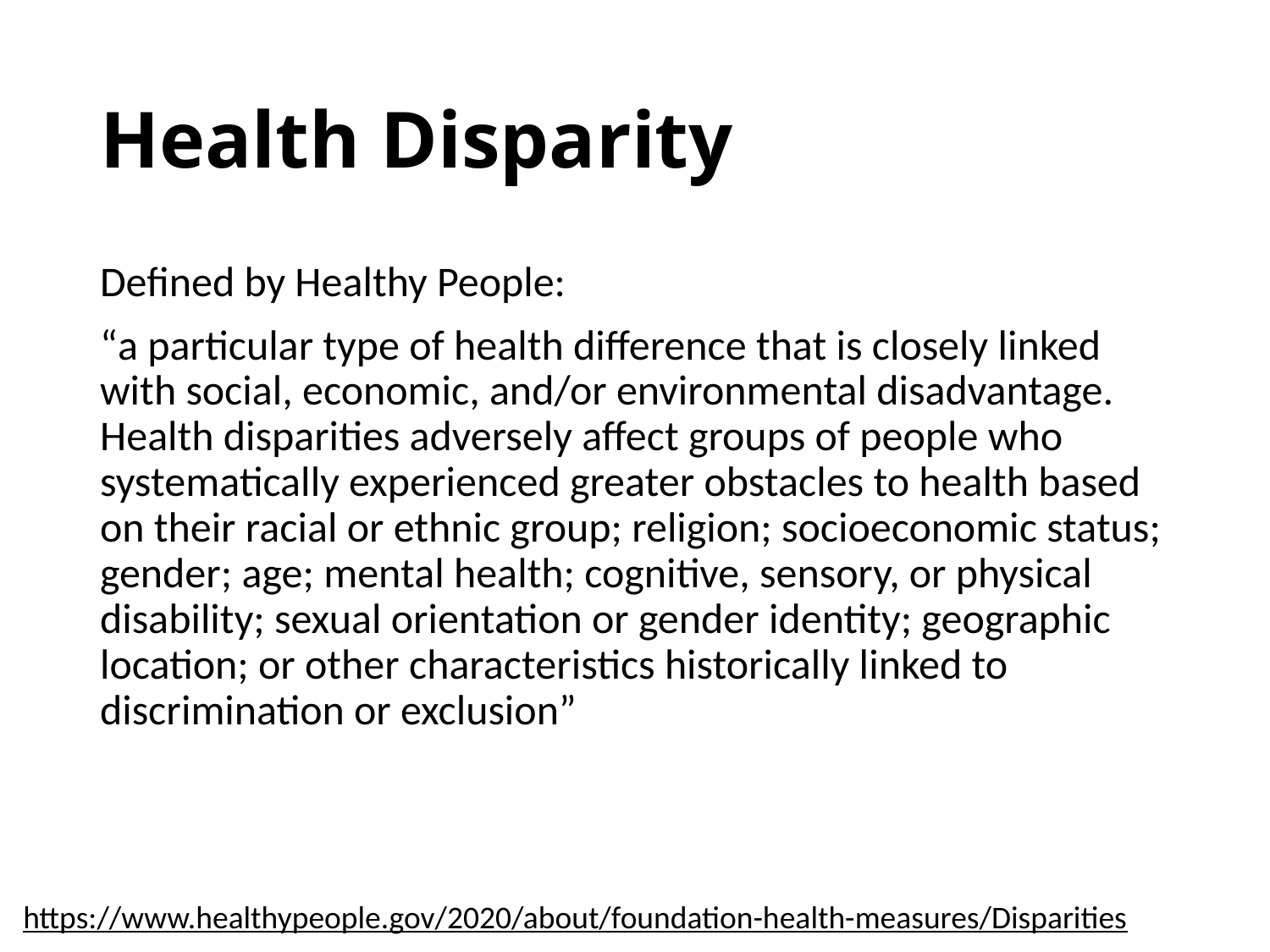

# Health Disparity
Defined by Healthy People:
“a particular type of health difference that is closely linked with social, economic, and/or environmental disadvantage.  Health disparities adversely affect groups of people who systematically experienced greater obstacles to health based on their racial or ethnic group; religion; socioeconomic status; gender; age; mental health; cognitive, sensory, or physical disability; sexual orientation or gender identity; geographic location; or other characteristics historically linked to discrimination or exclusion”
https://www.healthypeople.gov/2020/about/foundation-health-measures/Disparities

## Slide 6
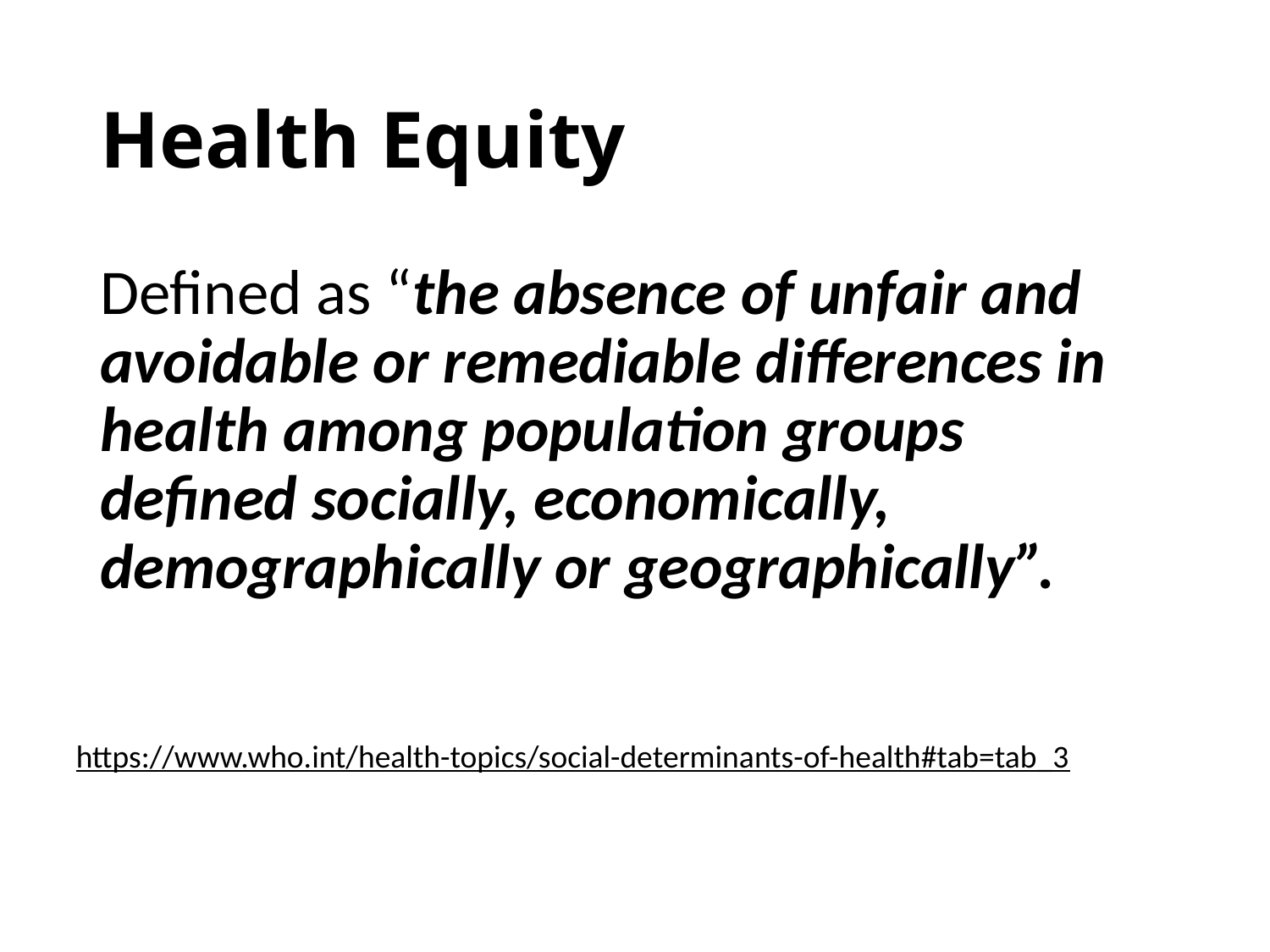

# Health Equity
Defined as “the absence of unfair and avoidable or remediable differences in health among population groups defined socially, economically, demographically or geographically”.
https://www.who.int/health-topics/social-determinants-of-health#tab=tab_3

## Slide 7
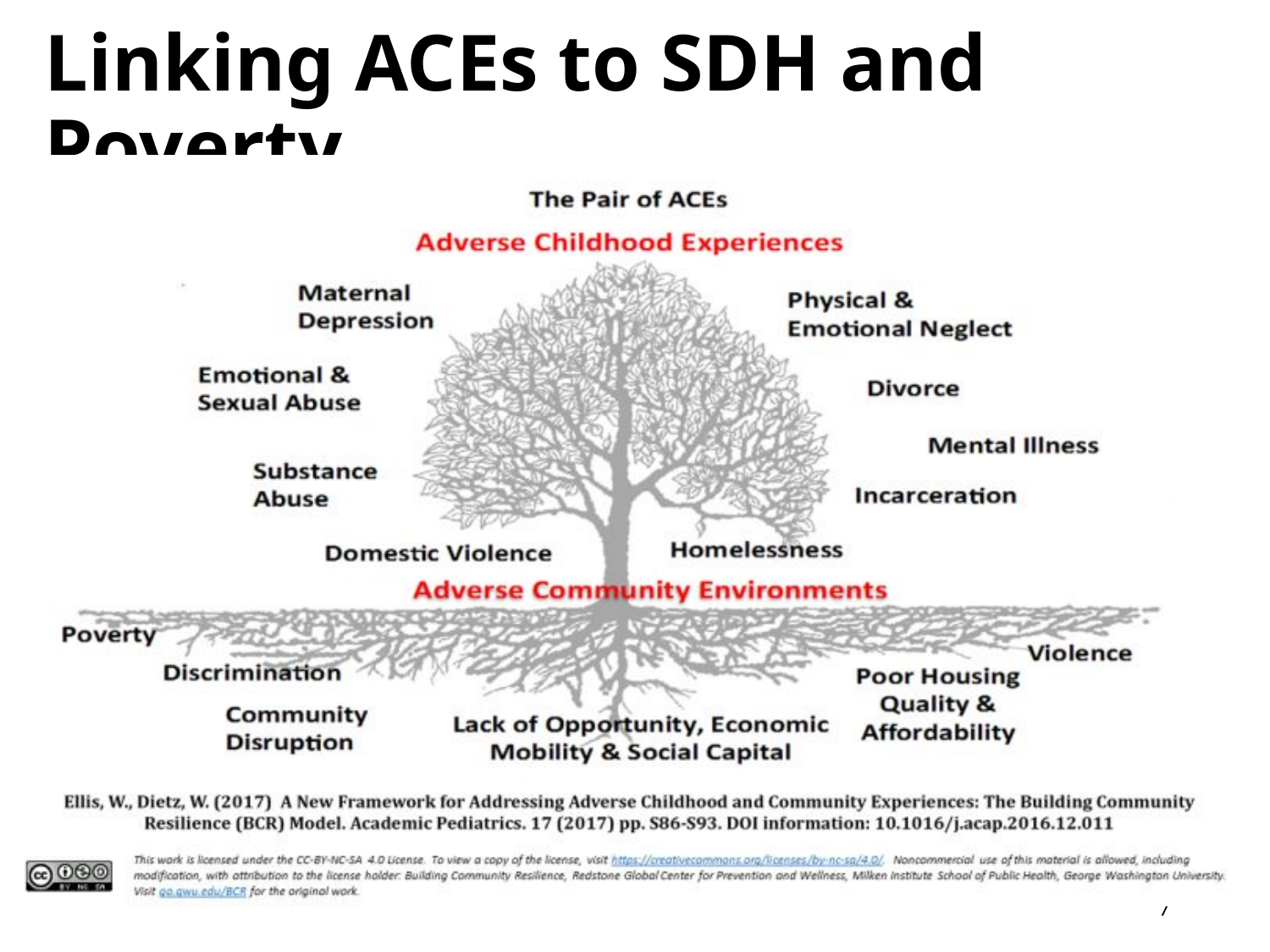

# Linking ACEs to SDH and Poverty
7

## Slide 8
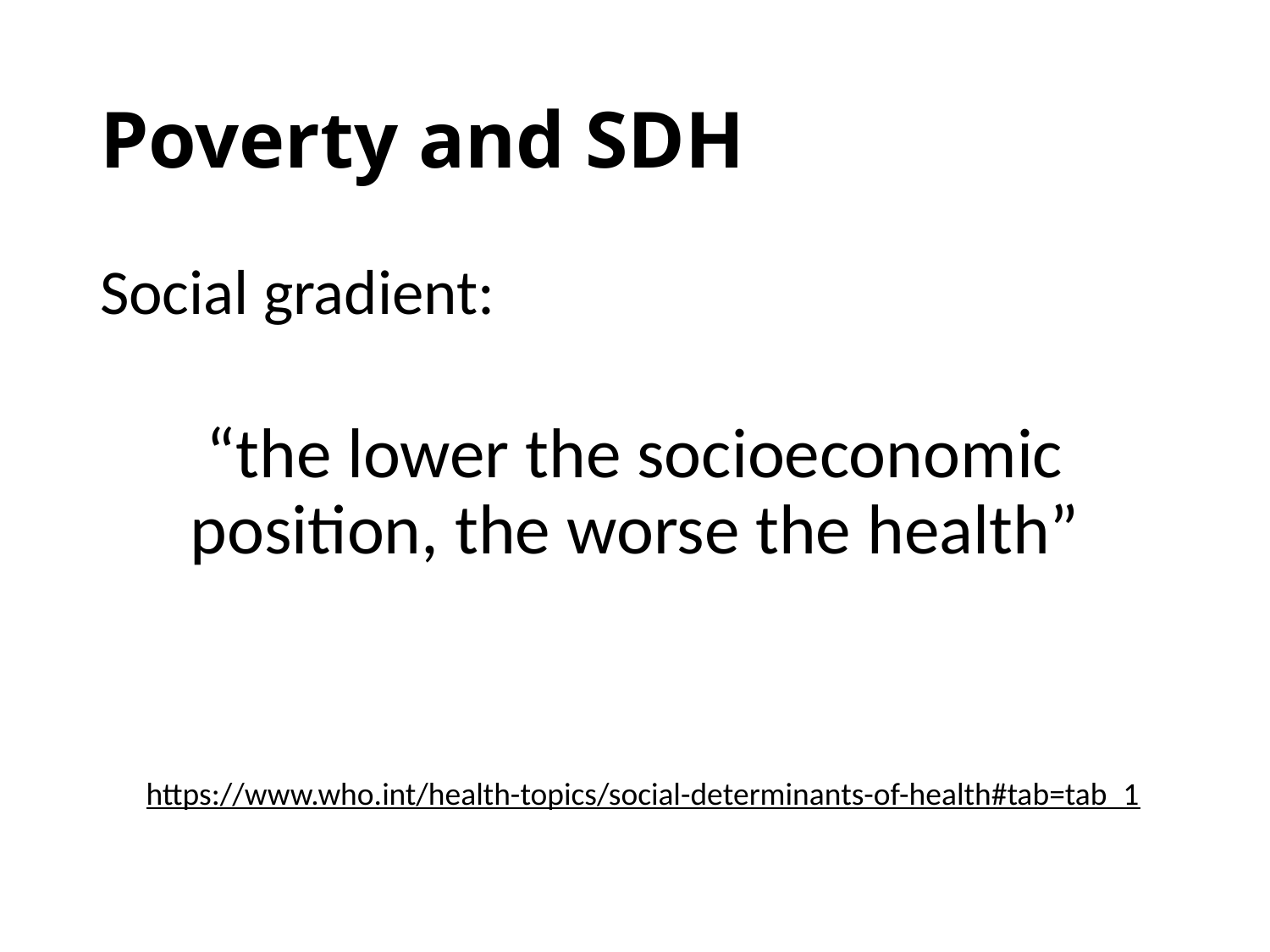

# Poverty and SDH
Social gradient:
“the lower the socioeconomic position, the worse the health”
https://www.who.int/health-topics/social-determinants-of-health#tab=tab_1

## Slide 9
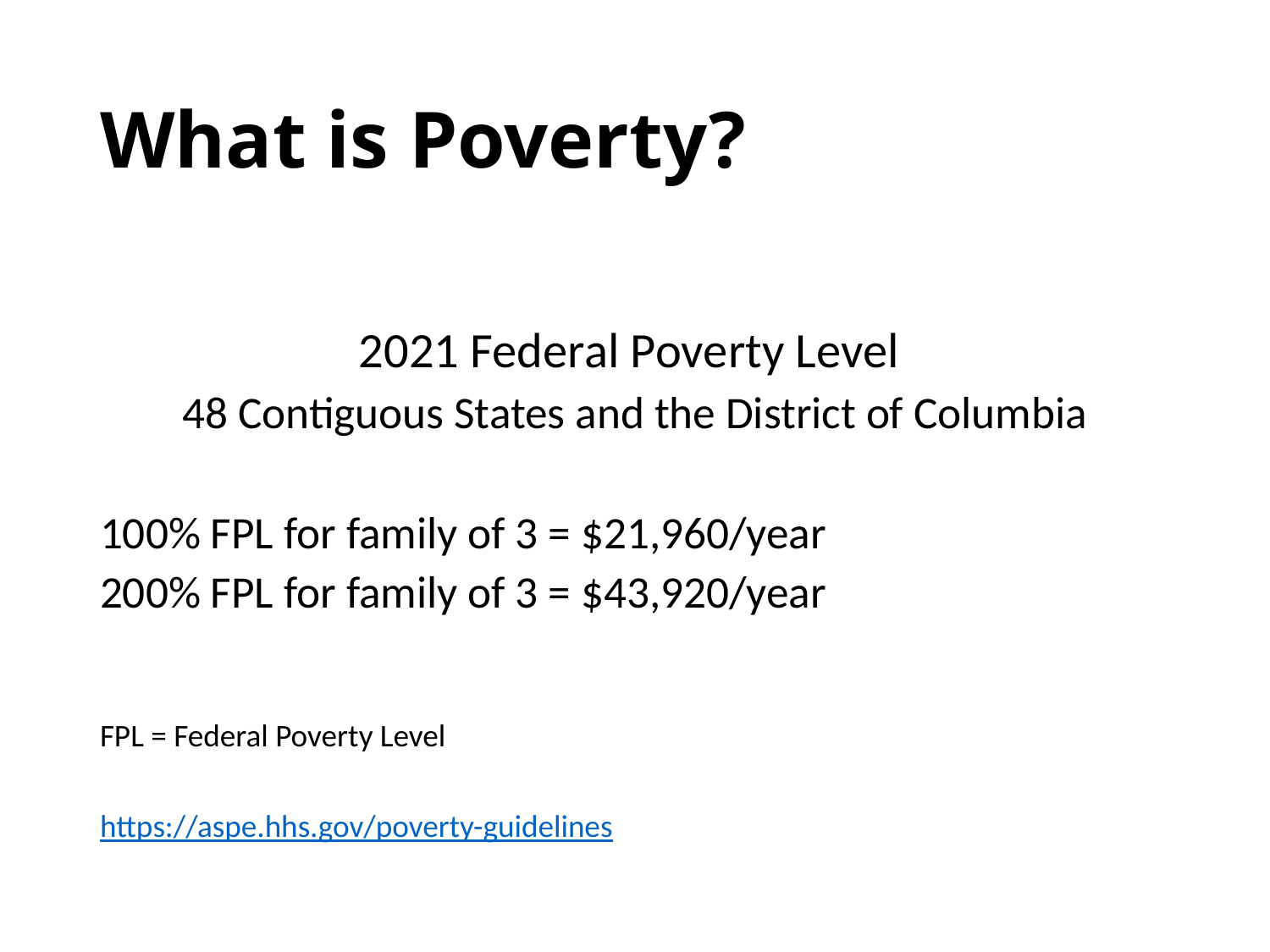

# What is Poverty?
2021 Federal Poverty Level
48 Contiguous States and the District of Columbia
100% FPL for family of 3 = $21,960/year
200% FPL for family of 3 = $43,920/year
FPL = Federal Poverty Level
https://aspe.hhs.gov/poverty-guidelines

## Slide 10
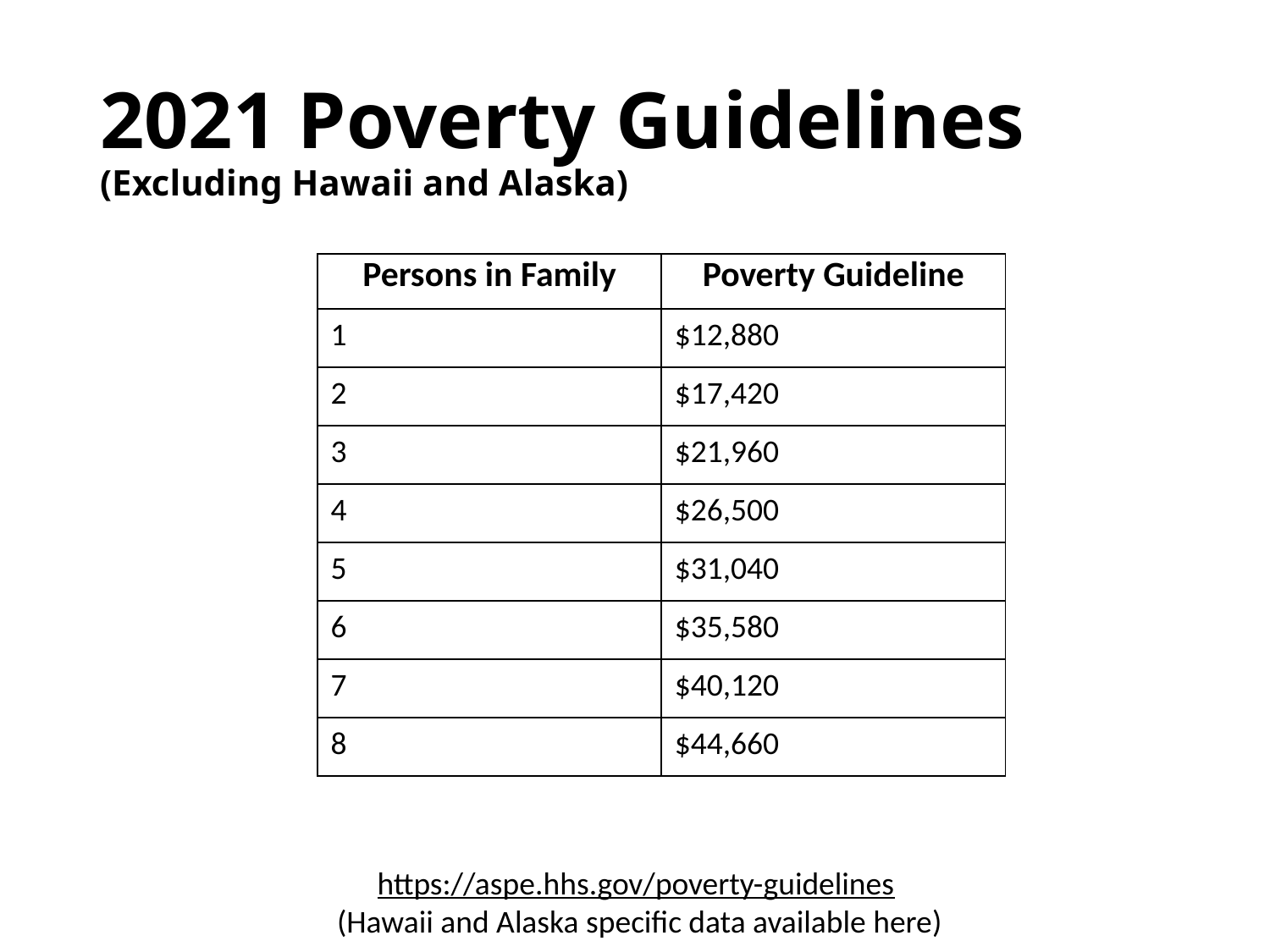

# 2021 Poverty Guidelines (Excluding Hawaii and Alaska)
| Persons in Family | Poverty Guideline |
| --- | --- |
| 1 | $12,880 |
| 2 | $17,420 |
| 3 | $21,960 |
| 4 | $26,500 |
| 5 | $31,040 |
| 6 | $35,580 |
| 7 | $40,120 |
| 8 | $44,660 |
https://aspe.hhs.gov/poverty-guidelines
(Hawaii and Alaska specific data available here)

## Slide 11
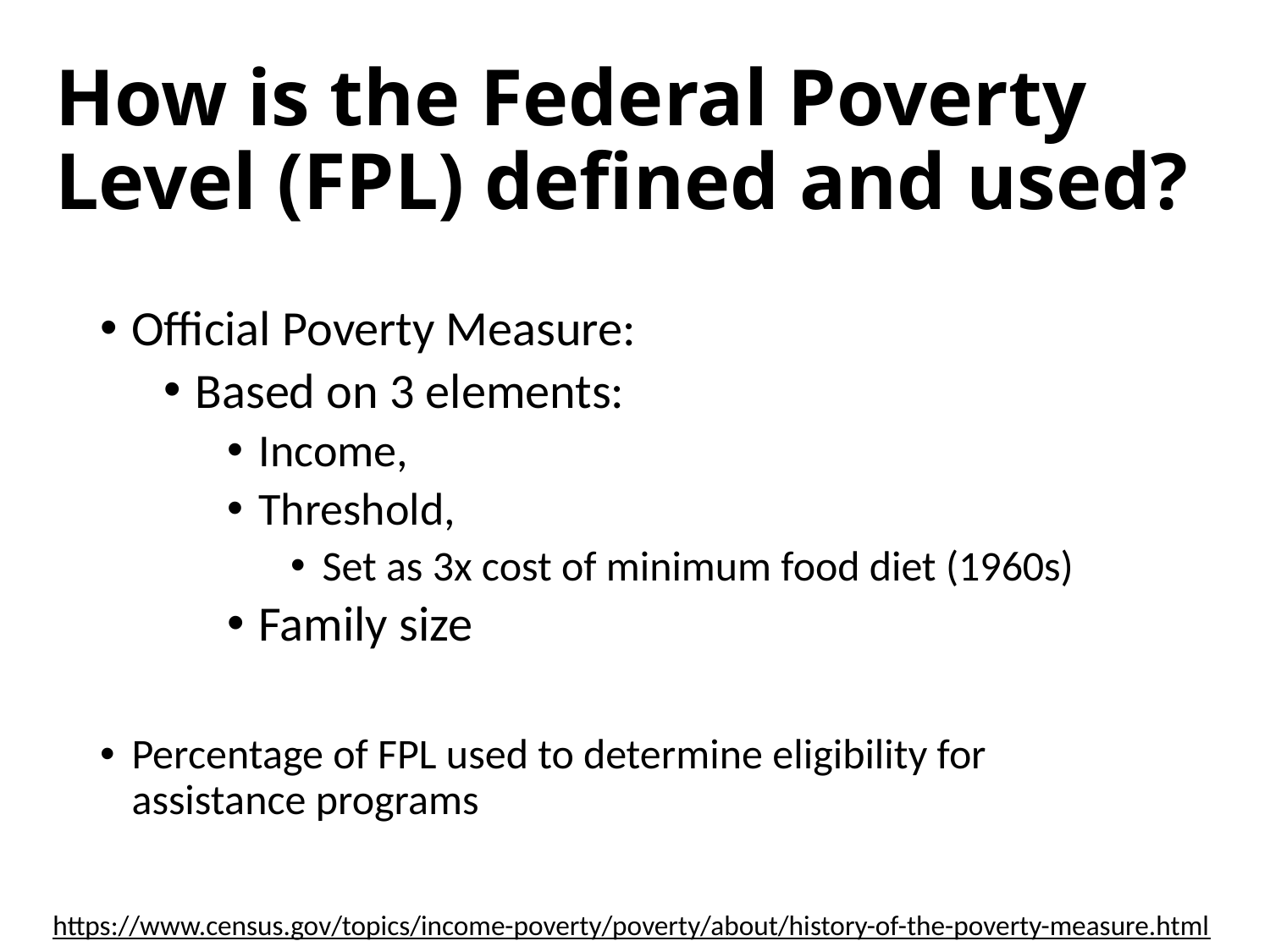

# How is the Federal Poverty Level (FPL) defined and used?
Official Poverty Measure:
Based on 3 elements:
Income,
Threshold,
Set as 3x cost of minimum food diet (1960s)
Family size
Percentage of FPL used to determine eligibility for assistance programs
https://www.census.gov/topics/income-poverty/poverty/about/history-of-the-poverty-measure.html

## Slide 12
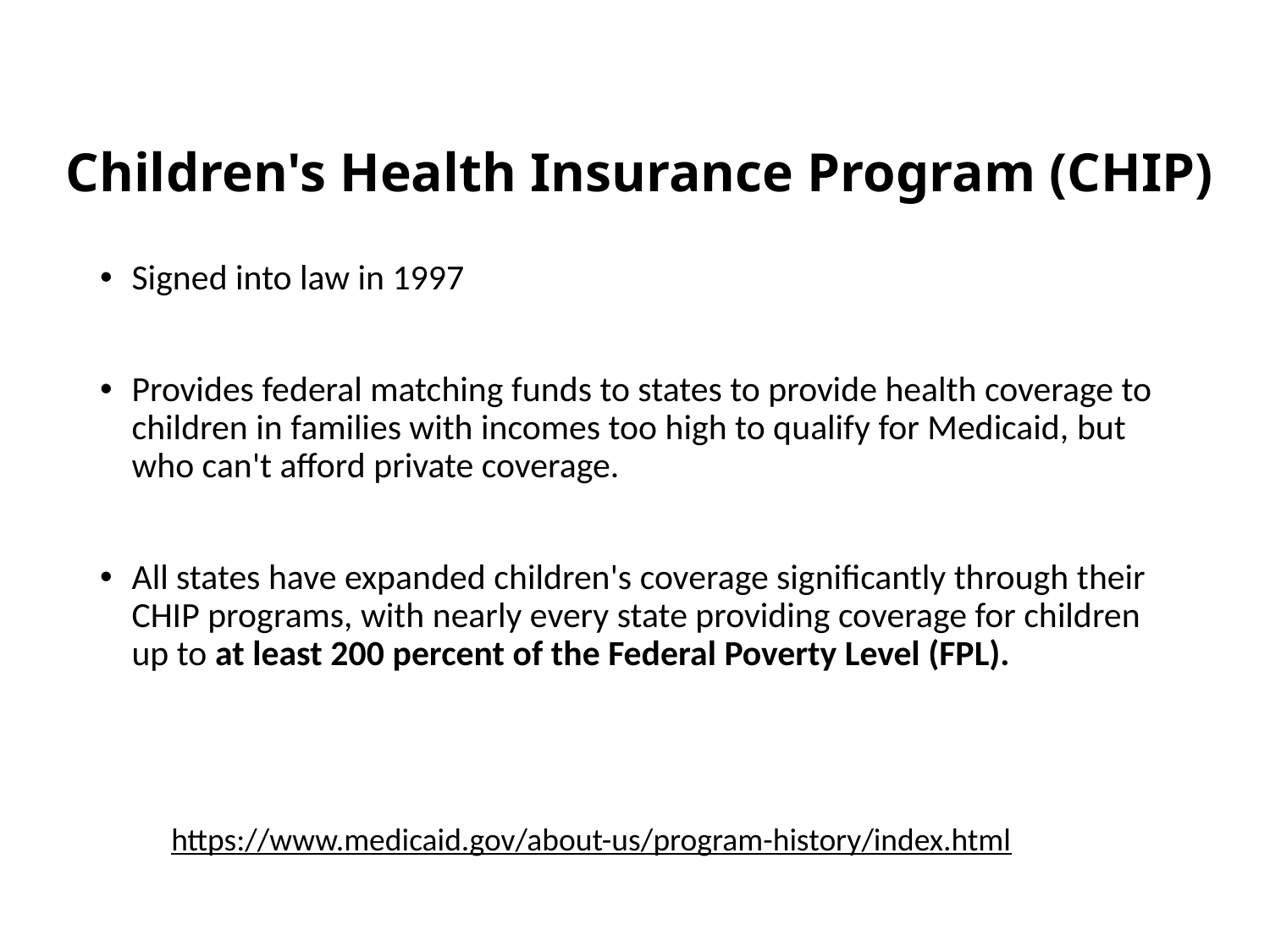

# Children's Health Insurance Program (CHIP)
Signed into law in 1997
Provides federal matching funds to states to provide health coverage to children in families with incomes too high to qualify for Medicaid, but who can't afford private coverage.
All states have expanded children's coverage significantly through their CHIP programs, with nearly every state providing coverage for children up to at least 200 percent of the Federal Poverty Level (FPL).
https://www.medicaid.gov/about-us/program-history/index.html

## Slide 13
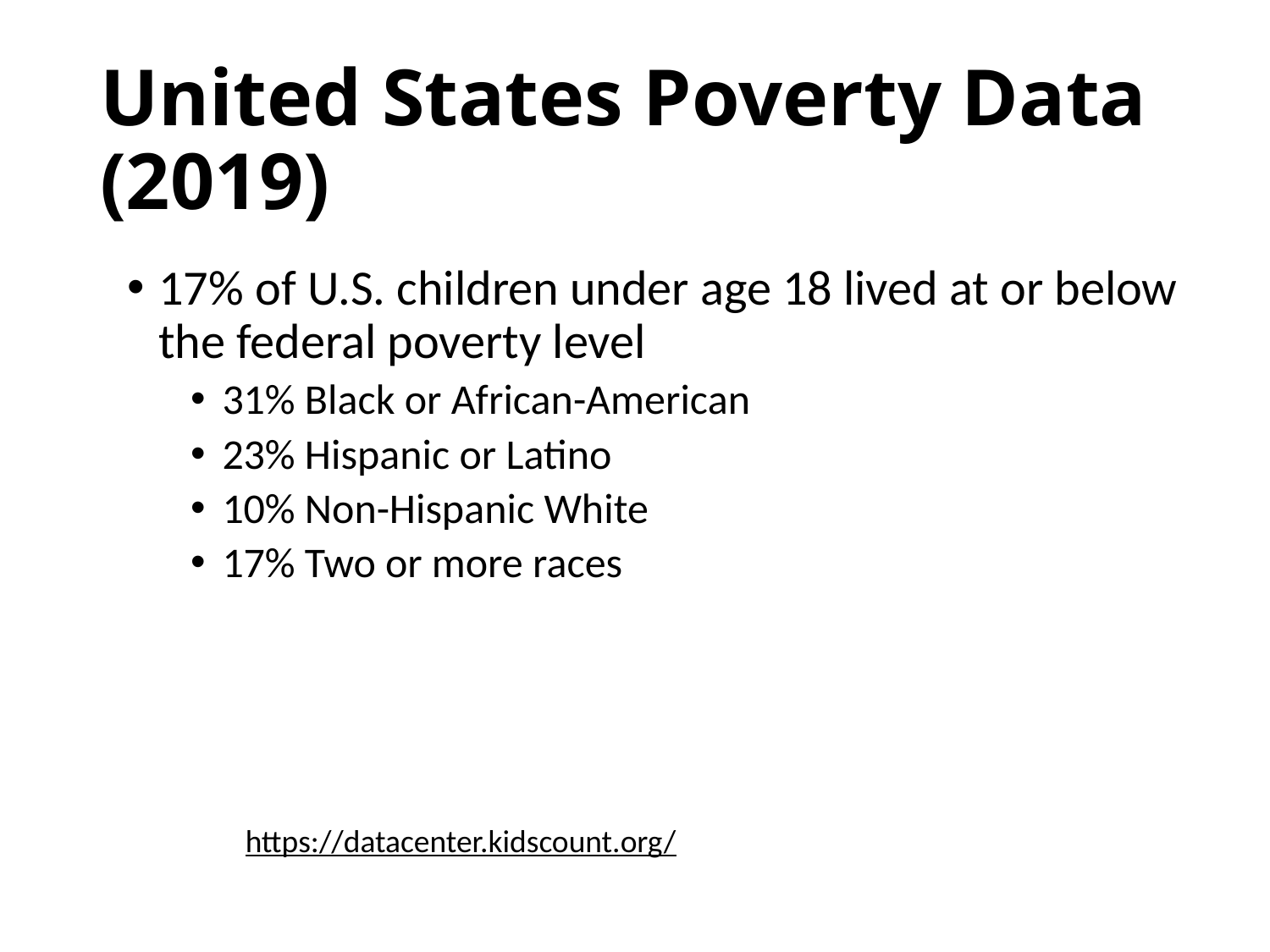

# United States Poverty Data (2019)
17% of U.S. children under age 18 lived at or below the federal poverty level
31% Black or African-American
23% Hispanic or Latino
10% Non-Hispanic White
17% Two or more races
https://datacenter.kidscount.org/

## Slide 14
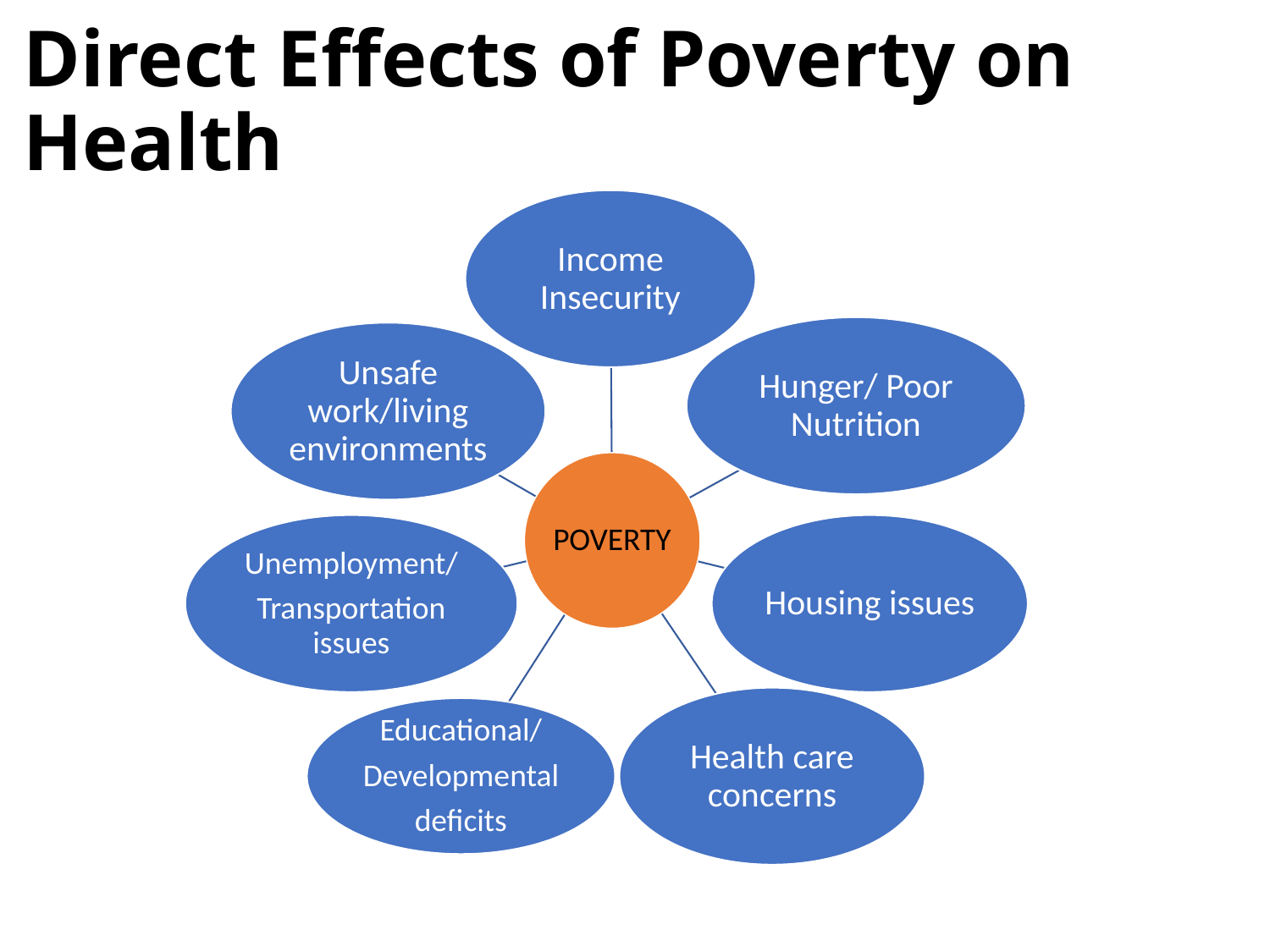

# Direct Effects of Poverty on Health

## Slide 15
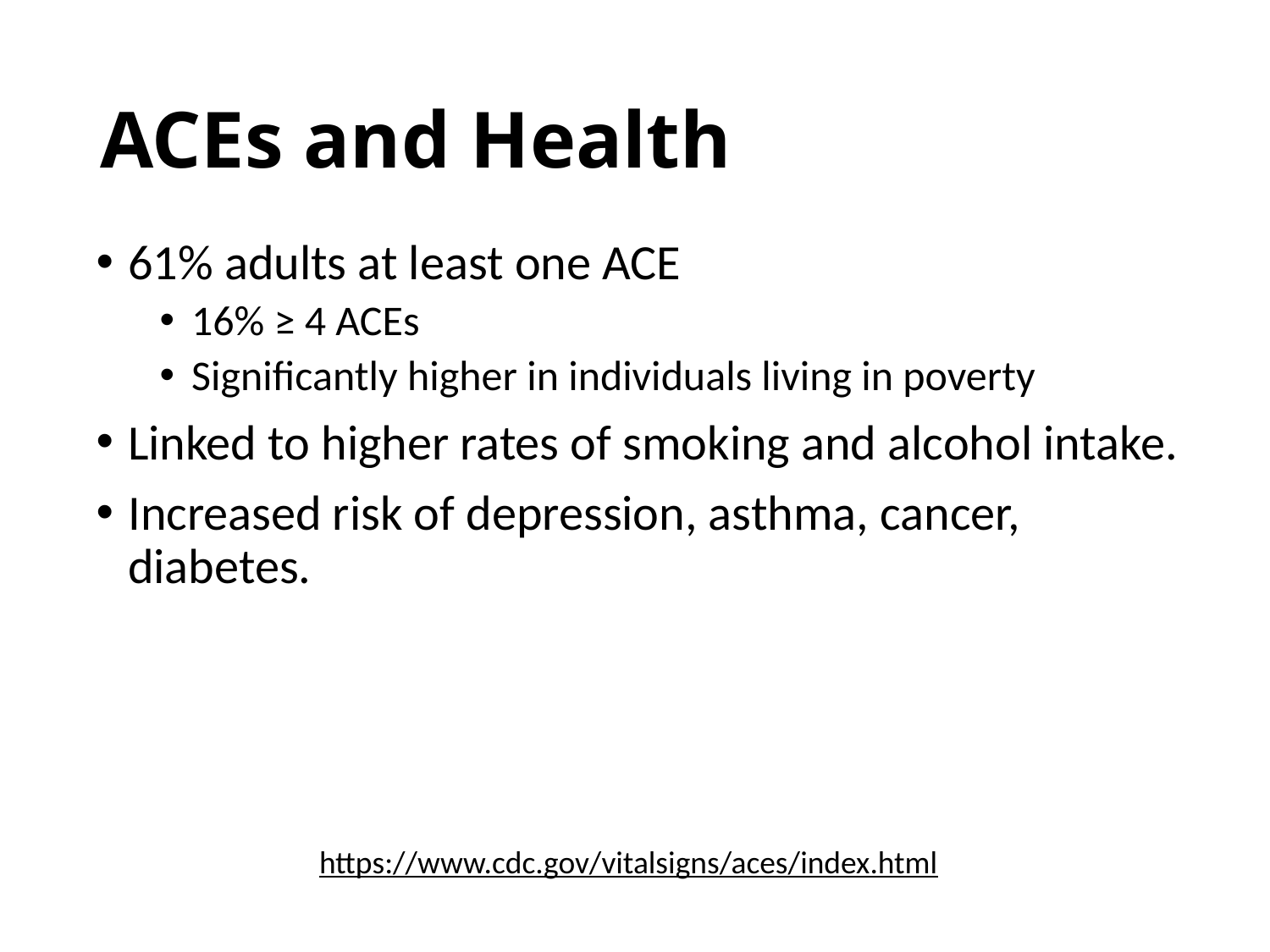

# ACEs and Health
61% adults at least one ACE
16% ≥ 4 ACEs
Significantly higher in individuals living in poverty
Linked to higher rates of smoking and alcohol intake.
Increased risk of depression, asthma, cancer, diabetes.
https://www.cdc.gov/vitalsigns/aces/index.html

## Slide 16
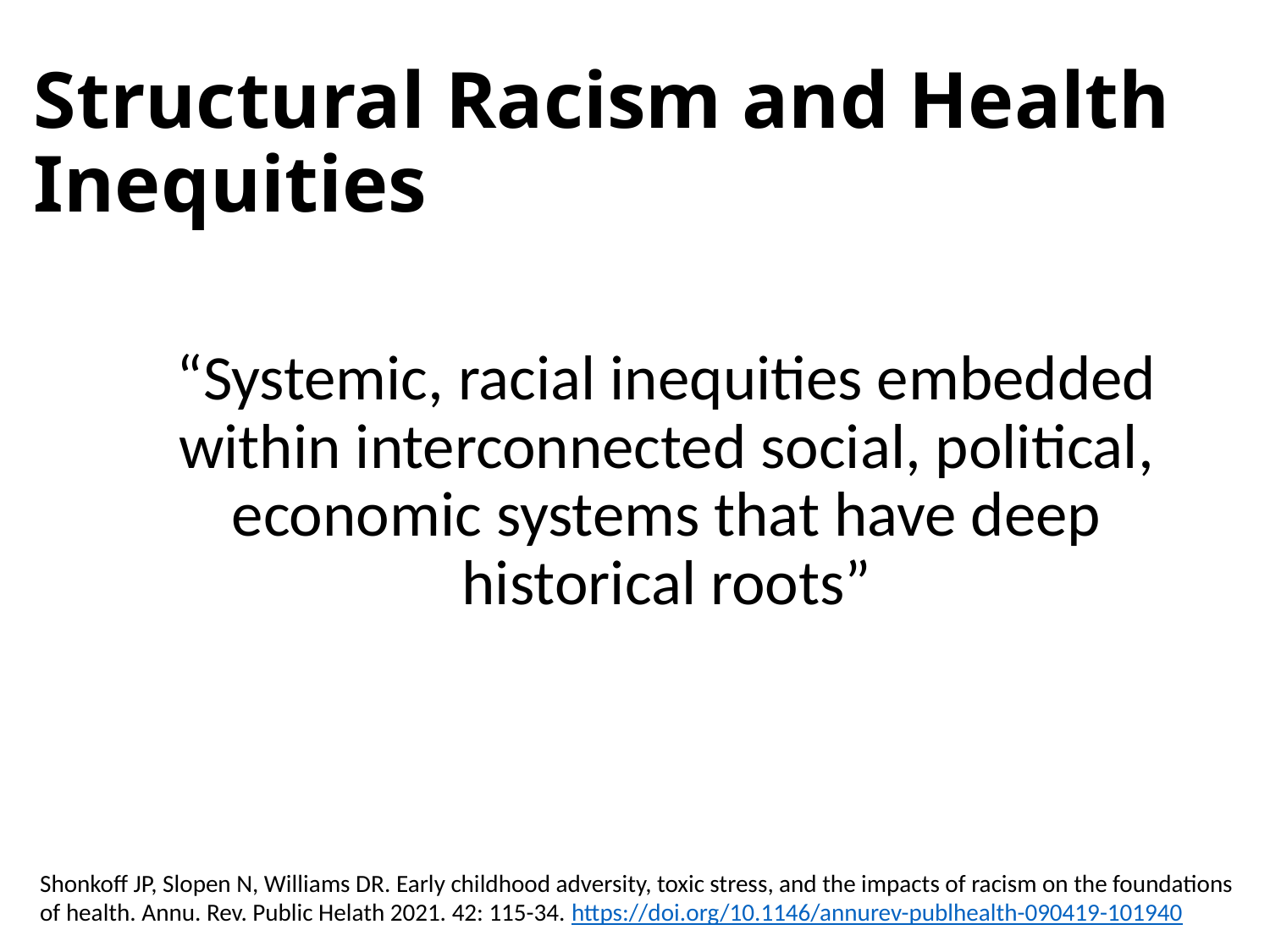

# Structural Racism and Health Inequities
“Systemic, racial inequities embedded within interconnected social, political, economic systems that have deep historical roots”
Shonkoff JP, Slopen N, Williams DR. Early childhood adversity, toxic stress, and the impacts of racism on the foundations of health. Annu. Rev. Public Helath 2021. 42: 115-34. https://doi.org/10.1146/annurev-publhealth-090419-101940

## Slide 17
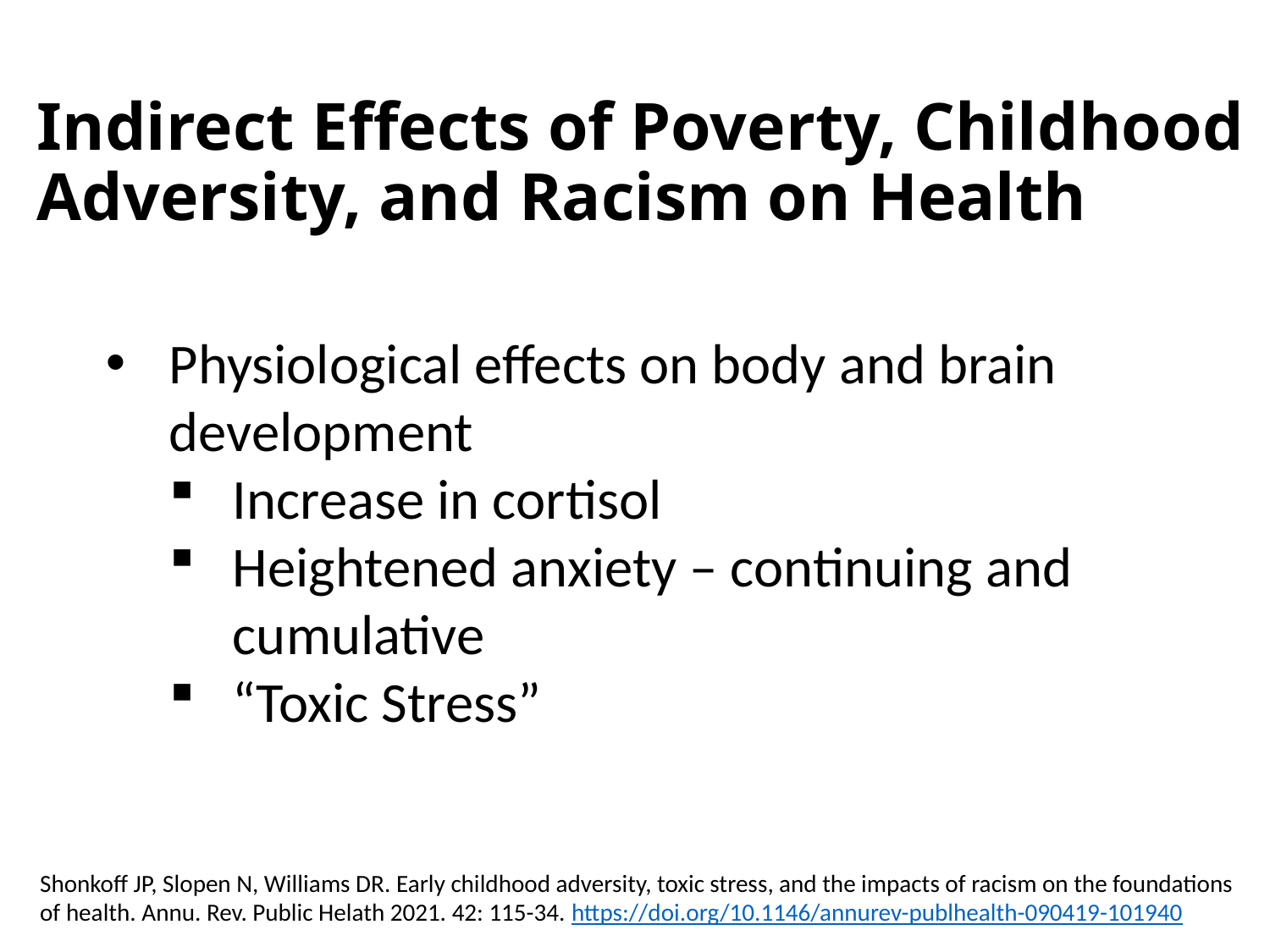

# Indirect Effects of Poverty, Childhood Adversity, and Racism on Health
Physiological effects on body and brain development
Increase in cortisol
Heightened anxiety – continuing and cumulative
“Toxic Stress”
Shonkoff JP, Slopen N, Williams DR. Early childhood adversity, toxic stress, and the impacts of racism on the foundations of health. Annu. Rev. Public Helath 2021. 42: 115-34. https://doi.org/10.1146/annurev-publhealth-090419-101940

## Slide 18
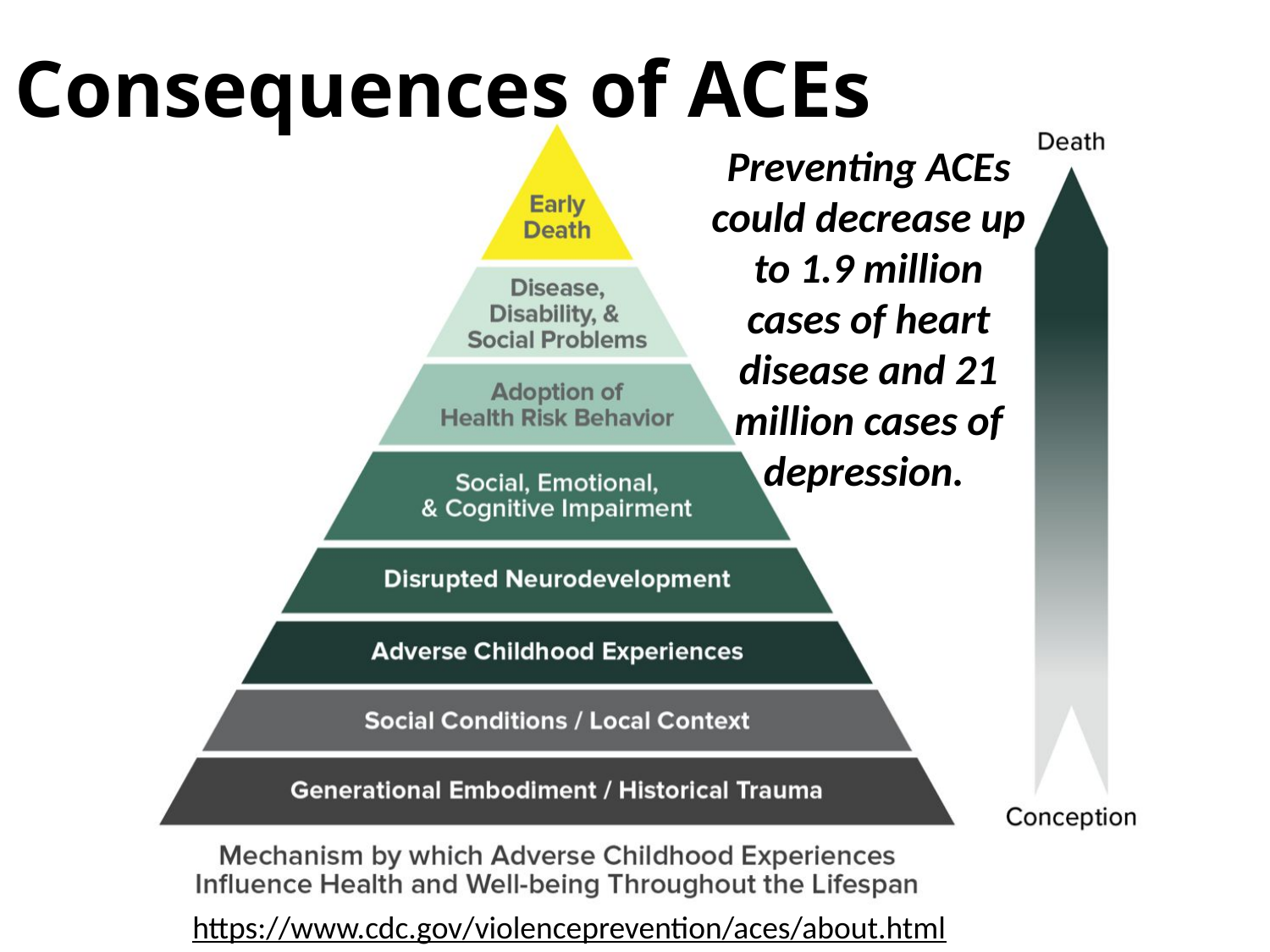

# Consequences of ACEs
Preventing ACEs could decrease up to 1.9 million cases of heart disease and 21 million cases of depression.
https://www.cdc.gov/violenceprevention/aces/about.html

## Slide 19
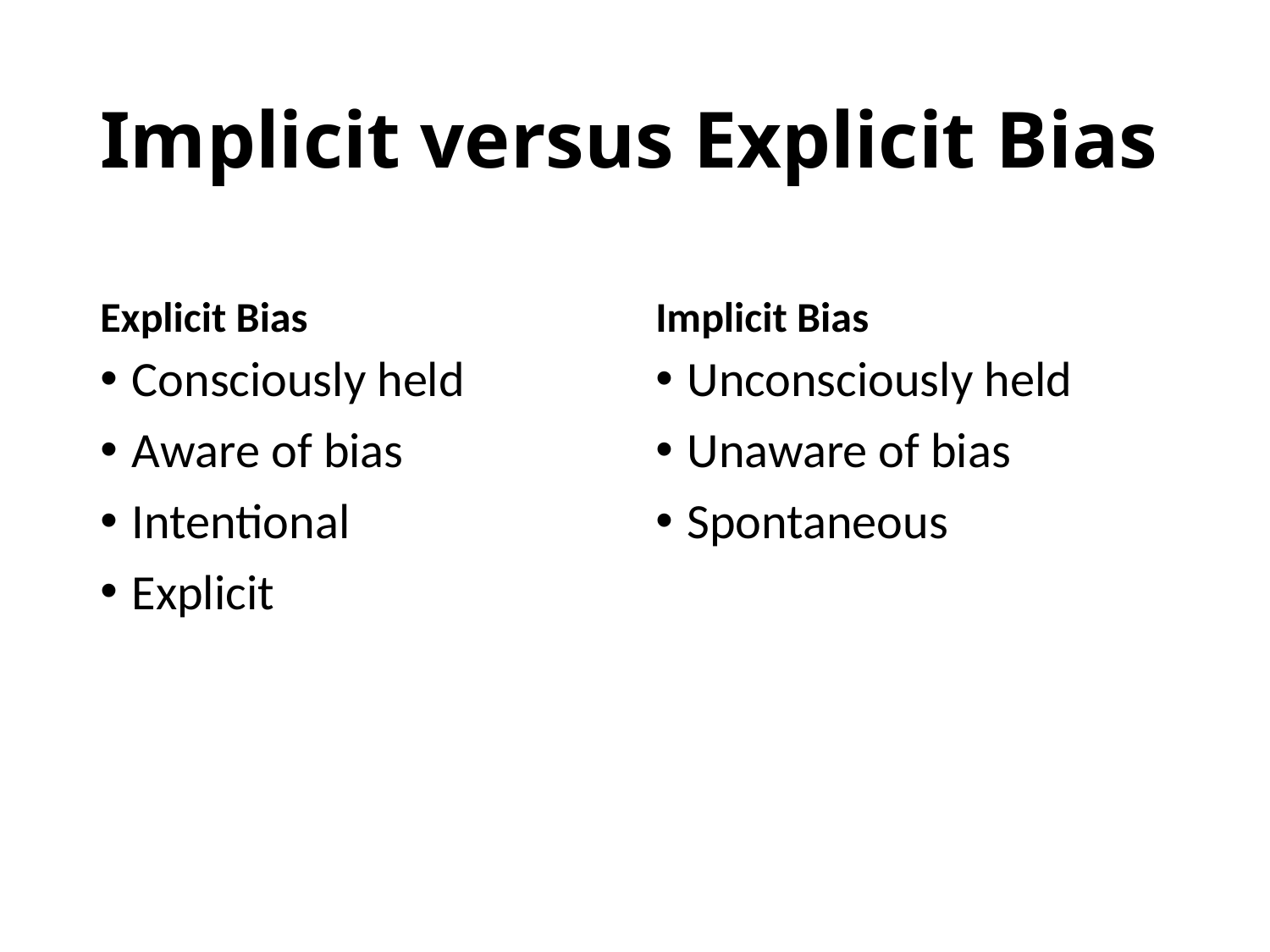

# Implicit versus Explicit Bias
Explicit Bias
Implicit Bias
Consciously held
Aware of bias
Intentional
Explicit
Unconsciously held
Unaware of bias
Spontaneous

## Slide 20
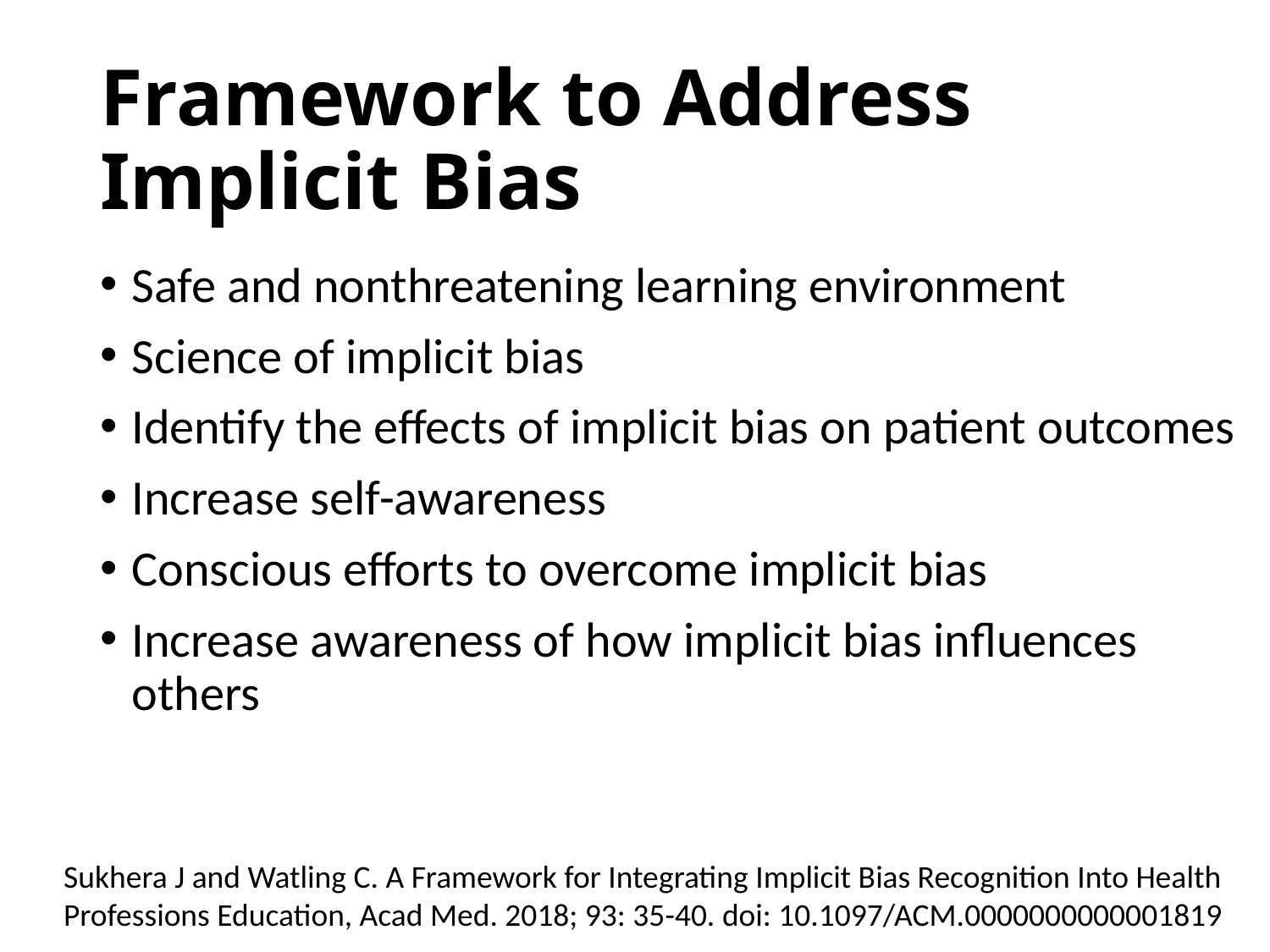

# Framework to Address Implicit Bias
Safe and nonthreatening learning environment
Science of implicit bias
Identify the effects of implicit bias on patient outcomes
Increase self-awareness
Conscious efforts to overcome implicit bias
Increase awareness of how implicit bias influences others
Sukhera J and Watling C. A Framework for Integrating Implicit Bias Recognition Into Health Professions Education, Acad Med. 2018; 93: 35-40. doi: 10.1097/ACM.0000000000001819

## Slide 21
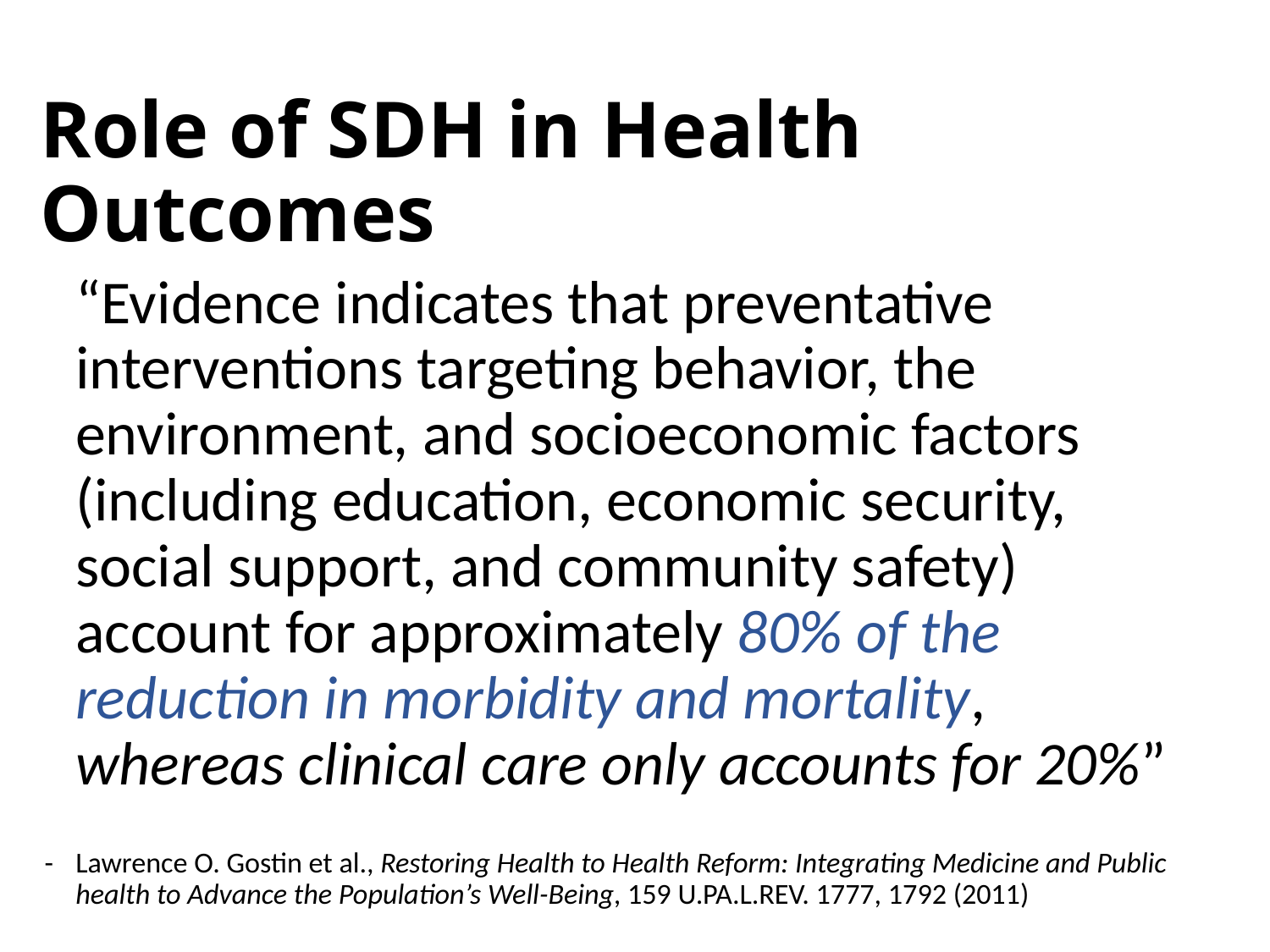

# Role of SDH in Health Outcomes
	“Evidence indicates that preventative interventions targeting behavior, the environment, and socioeconomic factors (including education, economic security, social support, and community safety) account for approximately 80% of the reduction in morbidity and mortality, whereas clinical care only accounts for 20%”
-	Lawrence O. Gostin et al., Restoring Health to Health Reform: Integrating Medicine and Public health to Advance the Population’s Well-Being, 159 U.PA.L.REV. 1777, 1792 (2011)

## Slide 22
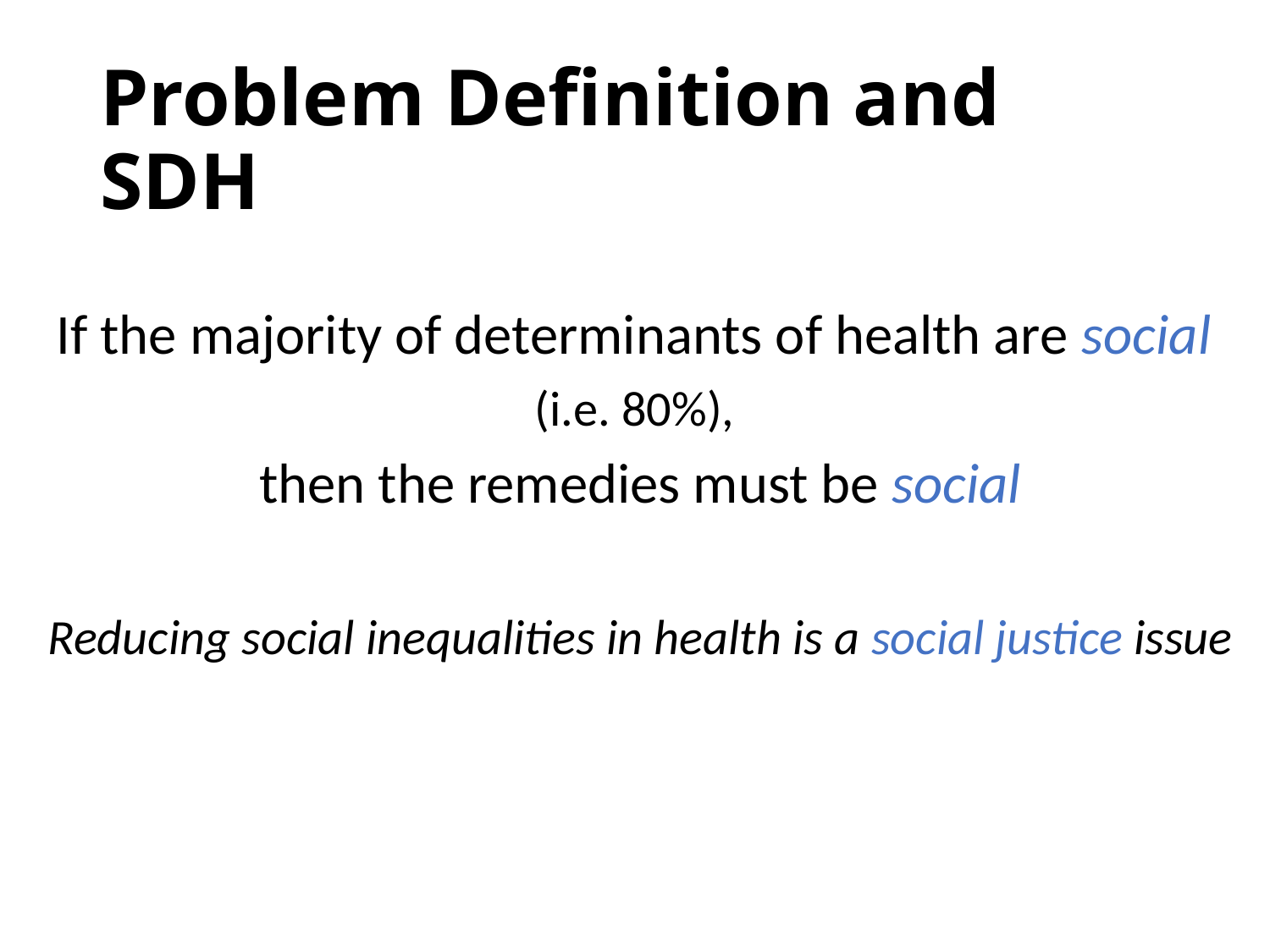

# Problem Definition and SDH
If the majority of determinants of health are social
(i.e. 80%),
then the remedies must be social
Reducing social inequalities in health is a social justice issue

## Slide 23
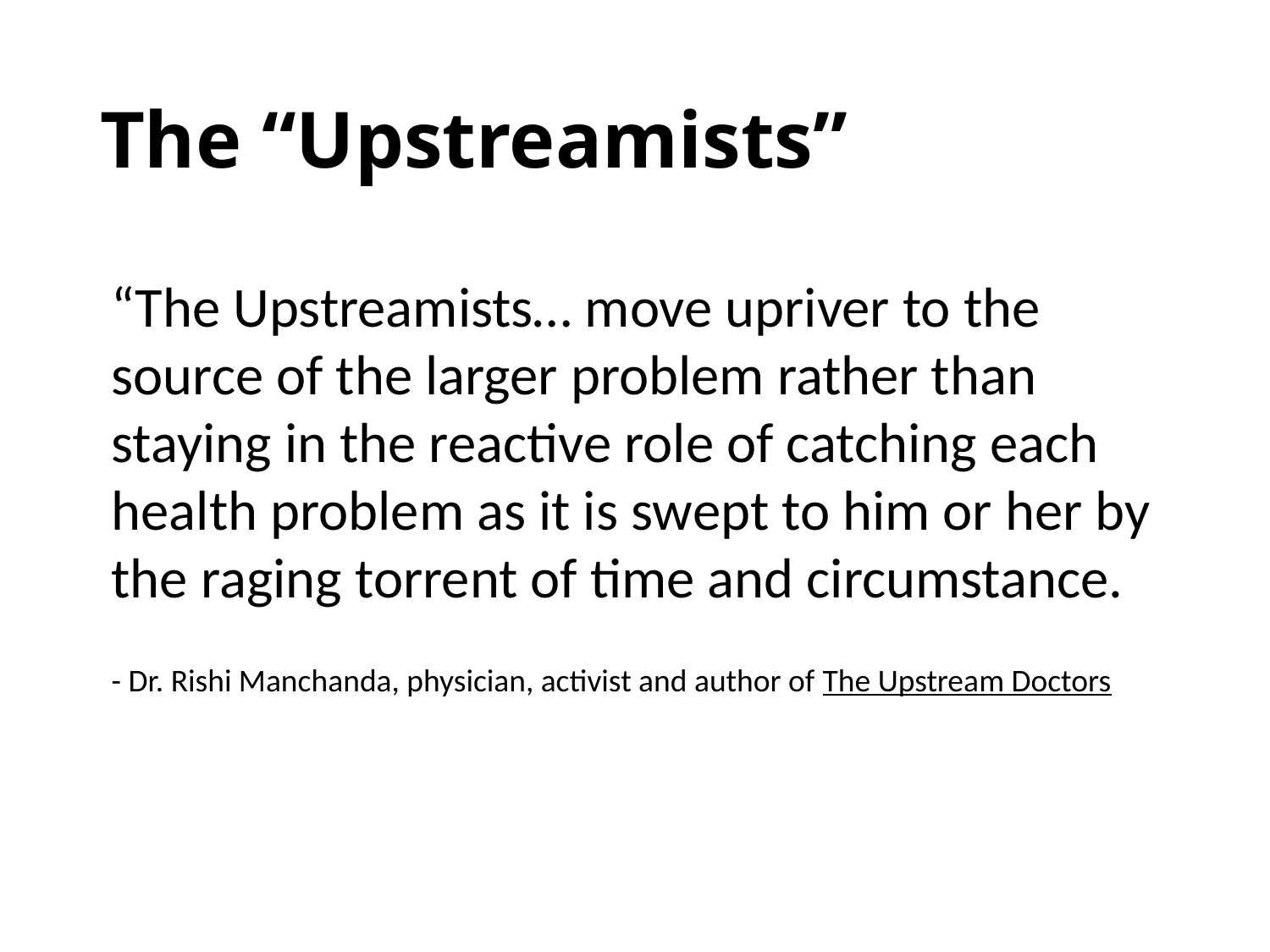

# The “Upstreamists”
“The Upstreamists… move upriver to the source of the larger problem rather than staying in the reactive role of catching each health problem as it is swept to him or her by the raging torrent of time and circumstance.
- Dr. Rishi Manchanda, physician, activist and author of The Upstream Doctors

## Slide 24
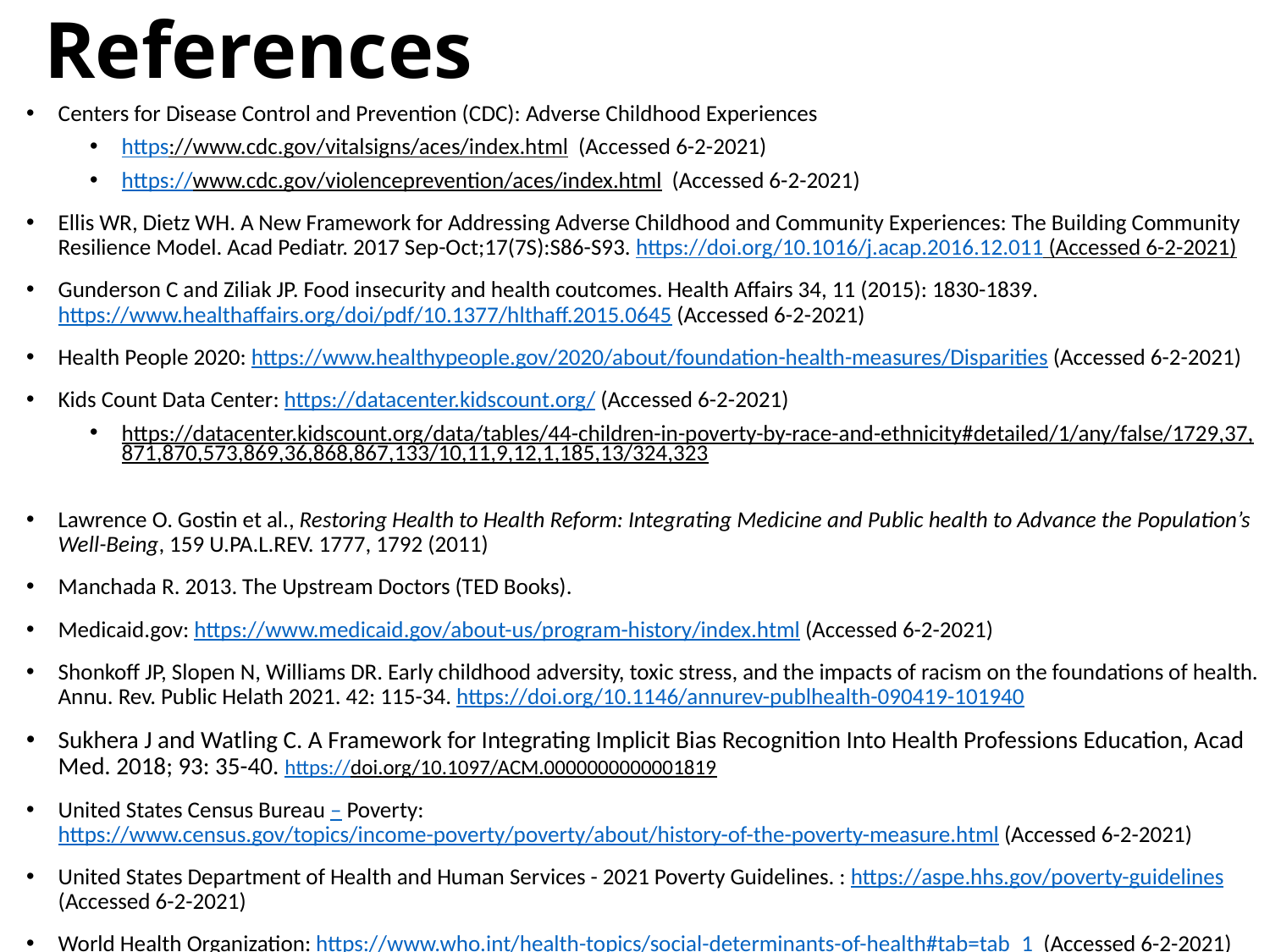

# References
Centers for Disease Control and Prevention (CDC): Adverse Childhood Experiences
https://www.cdc.gov/vitalsigns/aces/index.html (Accessed 6-2-2021)
https://www.cdc.gov/violenceprevention/aces/index.html (Accessed 6-2-2021)
Ellis WR, Dietz WH. A New Framework for Addressing Adverse Childhood and Community Experiences: The Building Community Resilience Model. Acad Pediatr. 2017 Sep-Oct;17(7S):S86-S93. https://doi.org/10.1016/j.acap.2016.12.011 (Accessed 6-2-2021)
Gunderson C and Ziliak JP. Food insecurity and health coutcomes. Health Affairs 34, 11 (2015): 1830-1839. https://www.healthaffairs.org/doi/pdf/10.1377/hlthaff.2015.0645 (Accessed 6-2-2021)
Health People 2020: https://www.healthypeople.gov/2020/about/foundation-health-measures/Disparities (Accessed 6-2-2021)
Kids Count Data Center: https://datacenter.kidscount.org/ (Accessed 6-2-2021)
https://datacenter.kidscount.org/data/tables/44-children-in-poverty-by-race-and-ethnicity#detailed/1/any/false/1729,37,871,870,573,869,36,868,867,133/10,11,9,12,1,185,13/324,323
Lawrence O. Gostin et al., Restoring Health to Health Reform: Integrating Medicine and Public health to Advance the Population’s Well-Being, 159 U.PA.L.REV. 1777, 1792 (2011)
Manchada R. 2013. The Upstream Doctors (TED Books).
Medicaid.gov: https://www.medicaid.gov/about-us/program-history/index.html (Accessed 6-2-2021)
Shonkoff JP, Slopen N, Williams DR. Early childhood adversity, toxic stress, and the impacts of racism on the foundations of health. Annu. Rev. Public Helath 2021. 42: 115-34. https://doi.org/10.1146/annurev-publhealth-090419-101940
Sukhera J and Watling C. A Framework for Integrating Implicit Bias Recognition Into Health Professions Education, Acad Med. 2018; 93: 35-40. https://doi.org/10.1097/ACM.0000000000001819
United States Census Bureau – Poverty: https://www.census.gov/topics/income-poverty/poverty/about/history-of-the-poverty-measure.html (Accessed 6-2-2021)
United States Department of Health and Human Services - 2021 Poverty Guidelines. : https://aspe.hhs.gov/poverty-guidelines (Accessed 6-2-2021)
World Health Organization: https://www.who.int/health-topics/social-determinants-of-health#tab=tab_1 (Accessed 6-2-2021)
