# Supplementary material for: Down to the Last Dollar: Utilizing a Virtual Budgeting Exercise to Recognize Implicit Bias
Source: MedEdPORTAL. 2021 Dec 6;17:11199. doi: 10.15766/mep_2374-8265.11199 (PMC8645532; doi:10.15766/mep_2374-8265.11199)
Supplement: Supplementary file 1 — Social Determinants of Health Lecture.pptxCase Scenario with Group Reflection Exercise.docxBudgeting Templates - Common Food Prices.xlsxExample of Budget - Chain Grocery Store.xlsxExample of Budget - Wholesale Grocery Store.xlsxFacilitator Guide.docxSession Evaluation.docx [file mep_2374-8265.11199-s001.zip › B. Case Scenario with Group Reflection Exercise.docx]

Appendix B: Case Scenario

You are a 26-year-old single parent of two children, Erica (10 years old) and Matthew (8 months old). You completed high school in Haiti, attended a teacher training program, and worked as a teacher’s assistant before coming to the U.S. You moved to this country with Erica in <***Insert year 3 years prior to current date***>. Your sister, an American citizen, sponsored you, and you are in the U.S. on refugee status. You have a green card and are able to work. You have worked a variety of jobs since arriving in the U.S., including clerical, food service, and janitorial, none of which were full-time. You have not had steady work in the last year and are currently unemployed. You live in a studio apartment in ________. You pay $800 per month in rent for a studio apartment.

You believe you are in good health, but you have not had a physical in several years. You and your children are covered by Medicaid health insurance. Erica suffers from epilepsy and obesity; she experiences both petit-mal and grand-mal seizures, despite medication. Matthew was born at 26 weeks and is diagnosed with chronic lung disease. He requires regular nebulizer treatments and frequent doctors’ visits due to his susceptibility to lung infections. You have no relationship with Matthew’s other parent who abandoned your family when he was just three weeks old.

Your current monthly income includes supplemental security income (SSI) that you receive for Matthew ($825) and SNAP benefits ($535). You receive an additional $200/month off the books by watching your neighbor’s children after school. You received temporary rental assistance (TRA) for six months since you were behind on your rent. You used the money to pay off the rent you owed your landlord. You applied for Section 8 housing and have been on the waiting list for nearly three years.

It is unlikely your income will change in future months so do your best not to put off costs until a later date.

Please create your monthly budget as follows:

1. Use the Overall Budgeting Template to track all expenses.
2. Use the Food Budget Template to track your total food expenses and then incorporate total amount into the Overall Budgeting Template.
3. Use the List of Common Food Prices to calculate the food expenses within the Food Budget Template. You may also search for costs on-line as well.

After completing your budget exercise, please complete your Group Reflection Assignment noted below.

**Reflection from Budgeting Exercise:**

What stereotypes or biases (positive and/or negative) did you come across during this budgeting exercise? Below are examples of potential sources of bias. Please address a minimum of 4 different biases.

- Food insecurities
- Poverty
- Budgeting
- Race/Ethnicity
- Gender
- Language
- Immigration status
- Geography

**Please submit one response per group.**
