# Supplementary material for: Down to the Last Dollar: Utilizing a Virtual Budgeting Exercise to Recognize Implicit Bias
Source: MedEdPORTAL. 2021 Dec 6;17:11199. doi: 10.15766/mep_2374-8265.11199 (PMC8645532; doi:10.15766/mep_2374-8265.11199)
Supplement: Supplementary file 1 — Social Determinants of Health Lecture.pptxCase Scenario with Group Reflection Exercise.docxBudgeting Templates - Common Food Prices.xlsxExample of Budget - Chain Grocery Store.xlsxExample of Budget - Wholesale Grocery Store.xlsxFacilitator Guide.docxSession Evaluation.docx [file mep_2374-8265.11199-s001.zip › F. Facilitator Guide.docx]

**Appendix F: Facilitator Guide for Budgeting Exercise**

**Overview of Budgeting Exercise:**

This is a small group, simulated, virtual (or in-person) budgeting exercise to expose students to the effects of poverty and food insecurities on health as well as to provide them the opportunity to reflect on individual biases regarding patients living in poverty and their healthcare adherence.

**Student Preparation:**

**Overview of Social Determinants of Health PowerPoint (30 minutes; Appendix A):**

This can be administered as a podcast with requirement to review in advance of this session or it can be added to the beginning of the session as a live lecture. We chose to record this as a podcast and have students review in advance of this session.

**Suggested Timeline:**

| **Activity** | **Setting** | **Time** |
| --- | --- | --- |
| Case Scenario Review | Virtual large group | 10 minutes |
| Breakout Small Groups | Virtual small group (3-4 students) | 45 minutes |
| Group Reflection in Small Groups | Virtual small group (3-4 students) | 15 minutes |
| Group Debrief | Virtual large group | 30 minutes |
| **Total** | | **1 hour and 40 minutes** |

**Background Information and Resources:**

1. DallaPiazza M, PadillaRegister M, Dwarakanath M, Obamedo E, Hill J, Soto-Greene ML. Exploring racism and health: an intensive interactive session for medical students. MedEdPORTAL. 2018;14:10783. [https://doi.org/10.15766/mep_2374- 8265.10783](https://doi.org/10.15766/mep_2374-%208265.10783)
   1. Rutgers New Jersey Medical School students received this session during their preclerkship years.
      1. Review the CHARGE^2^ and INTERRUPT toolkits to mitigate bias.
2. **Supplemental Nutrition Assistance Program (SNAP)**
   1. **Eligibility:** Generally if your net monthly income is at or below the Federal Poverty Level
      1. [**https://www.fns.usda.gov/snap/recipient/eligibility**](https://www.fns.usda.gov/snap/recipient/eligibility)
   2. **Application:** Each state has its own application form.
      1. [**https://www.fns.usda.gov/snap/state-directory**](https://www.fns.usda.gov/snap/state-directory)
   3. **Immigration status**
      1. **Non-citizens must meet one of the following criteria:**
         - Lives in US for at least 5 years (exception if refugee status)
         - Receiving disability-related assistance
         - Children < 18 years of age
         - <https://www.fns.usda.gov/snap/eligibility/citizen/non-citizen-policy>
3. **Special Supplemental Nutrition Program for Women, Infants, and Children (WIC)**
   1. Supplemental foods and nutrition education for pregnant and postpartum women as well as infants and children up to 5 years old.
   2. <https://www.fns.usda.gov/wic>
   3. Application Process: <https://www.fns.usda.gov/wic/who-gets-wic-and-how-apply>
4. **Supplemental Security Income (SSI) for Children**
   1. <https://www.ssa.gov/benefits/disability/apply-child.html>
   2. Social Security’s definition of disability:
      1. “The child must have a physical or mental condition(s) that very seriously limits his or her activities; **and**
      2. The condition(s) must have lasted, or be expected to last, at least 1 year or result in death. A state agency makes the disability decision. They review the information you give us. They will also ask for information from medical and school sources and other people familiar with the child’s condition(s).”
5. **Immigrants and Medicaid**
   1. **“Qualified non-citizens” are eligible for Medicaid**
      1. Examples include lawful permanent residents/green cardholders, asylees, refugees, Cuban/Haitian entrants, victims of trafficking, federally recognized Indian tribe or American Indian born in Canada (refer to <https://www.healthcare.gov/immigrants/lawfully-present-immigrants/> for full list).
      2. 5-year waiting period for Green Card Holders/Lawful Permanent Residents
         - **Exceptions:** Refugees and Asylees or Lawful Permanent Residents who were refugees/asylees.
6. **Green Card Eligibility Categories**
   1. Sponsored through Family, Employment, Refugee or Asylee Status, Human Trafficking Victims, Victims of Abuse, and other categories.
      1. <https://www.uscis.gov/green-card/green-card-eligibility-categories>
   2. Application Process: <https://www.uscis.gov/green-card/how-to-apply-for-a-green-card>
7. **Adverse Childhood Experiences and Poverty**
   1. Nearly 50% of all American children have experienced at least 1 ACE
      1. Children of color at highest risk
      2. Children in the poorest families and communities show the greatest risk
      3. African American, American Indian and Hispanic children are more likely to live in high-poverty areas
      4. Higher prevalence of poverty, unemployment, and food insecurity
      5. Ellis WR and Dietz WH. A new framework for addressing Adverse Childhood and Community Experiences: The Building Community Resilience Model. *Academic Pediatrics*, 2017: 17 (7) S86-S93. <https://doi.org/10.1016/j.acap.2016.12.011>

**Implementation of the Budgeting Exercise:**

1. *Case Scenario (10 minutes):*

The case scenario provided to the student highlights a 26-year-old single parent of two children, ages 10 years and 8 months old. The facilitator reads the case to all students at the start of the session and then reviews the outline of the session, tasking the students to create a monthly budget based on the designated income. Students are provided the following documents in an Excel spreadsheet shared in a virtual platform:

- Case Scenario (Appendix B),
  1. Social Security amount received was the maximum allowable noted.
     1. <https://www.ssa.gov/benefits/disability/apply-child.html>
  2. Supplemental Nutrition Assistance Program (SNAP) <https://www.nj.gov/humanservices/njsnap/about/njsnap/>
- The maximum monthly benefit for 10/1/2020 through 9/30/2021 (3 persons in household) was utilized.
- Note there is currently a 15% increase during COVID-19 pandemic through 9/2021, which we have not included for this budgeting exercise as it is a temporary increase; this is the same amount across all states.
- Budget Form: Includes housing, utilities, telephone, food, cleaning products, personal care, transportation, and others (Appendix C – Spreadsheet 1),
- Food Budget Form: Includes a sample of foods/items they may use (Appendix C – Spreadsheet 2),
- List of Common Food Prices: Comparison of grocery cost from local food store, chain grocery store, and membership-only warehouse (Appendix C – Spreadsheet 3).

Student instructions are provided within the Overall Budget Form and Food Budget Form. For the List of Common Food Prices, students can also search for current prices from the internet based on the grocery stores surrounding their location.

1. *Breakout Groups (45 minutes):*

Students are randomly assigned into groups of 3-4 via breakout groups utilizing a virtual platform (could also be done in-person in same format). Each group has an on-line “shared” document with all of the above information, allowing each group to work independently and be able to view their own progress in real time. For example, if there are 5 groups of 3 students, each group has a separate budgeting form to complete which all members of the group can review via a shared online document. The facilitator checked in virtually with each breakout group and answered questions as needed.

**Overall Budget Form:** Please see Table 1 below for common questions/challenges that students may raise during the exercise in regards to the Overall and Food Budgets.

**Food Budget Form:** The list of food items are only examples. Students can edit/add/remove items as they see fit. Discuss potential for breakfast/lunch being provided at older child’s school, which would alleviate the need for 2 meals/day x 5 days each week while in school. In COVID, this created a crisis for many families; schools and local community centers quickly mobilized to provide these meals for children. Summer and other vacations can place a hardship on families reliant on school meals. Many cities/counties provide summer lunch programs to help offset this as well. Types of foods purchased is often purely based on cost, which highlights that higher carbohydrate, calorie and sugar dense products tend to be cheaper, more filling, and have longer shelf lives than fresh fruits, vegetables, healthy meats, etc. The cost of fast food relative to preparing a meal at home also can be discussed; recognize that it may be cheaper to purchase fast food than prepare a fresh meal.

Please refer to Table 1 below for common questions regarding the budgeting exercise. Please refer to Appendices D and E for examples of the overall and food budget templates utilizing a chain grocery store versus the wholesale grocery store. As you review the two examples, you will note that utilizing the wholesale grocery store saves ~$100 per month in food costs. However, the SNAP benefits can only be used for food (non-prepared items) and thus the family would not be able to utilize this in other areas of their budget. In addition, using the wholesale store, the parent would likely need a taxi or car share due to the distance and bulk of food bought, especially with two children, which adds significant cost to a roundtrip fare. The annual membership fee also needs to be accounted for in the budget.

1. *Group Reflection Exercise (15 minutes):*

At ~45 minutes, all groups complete the reflection assignment and submit it to the online learning management system, Canvas. Each group submits one response.

- What stereotypes or biases (positive and/or negative) did you come across during this budgeting exercise? Include food insecurities, poverty, budgeting, race/ethnicity, gender, language, immigration status, and geography. Students should reflect on a minimum of 4 different types of biases (Appendix B).

Please refer to Table 2 below for examples of biases identified.

1. *Large group debrief (30 minutes):*

After the group reflections are completed, all students return to the larger group where the facilitator debriefs with the students. Each group shares components of their group reflection. Facilitator reviews several next step or “what if” questions.

**Overall Debrief/Discussion:**

- What was hardest thing you noted in solving the budget?
  - Food
    - Quantifying how much children need over course of month
    - Utilizing restrictions with food assistance programs
    - Types of foods selected
    - Which grocery store to use? Local in walking distance vs chain grocery (further away) vs. wholesale
      - Including cost of transportation
      - Accounting for parent traveling with two children
      - Accounting for storage of bulk items
    - Balancing Non-Food Expenses
      - Decide which items are essential:
        - Cable/internet

Discuss COVID pandemic and need for virtual learning

If family did not have internet, they would not have access to education

- - - - - Hygiene products

Cost of tampons/pads vs menstrual cups (ease of use)

- - - - - Diapers

Cloth diapers versus disposable

Reminder that family does not have washer/dryer at home; cost of laundry for cloth diapers.

- What types of items were you unable to purchase with the given budget?
  - Fresh fruits/vegetables
  - Higher cost meats
  - Hygiene products
  - Clothing for the children
  - School expenses – backpack, supplies
  - Cable and/or internet
- What types of food did you purchase?
  - Discussion on lower cost items often being high carbohydrate, processed, added sugar, with higher shelf lives and more filling.
  - Fast food may be cheaper than preparing a meal
- Is there any funds left for “fun” activities such as going to the movies?
  - Rarely an option as cannot meet even the basic necessities on this budget.
  - Think of “free” options like parks, library, etc., but also in perspective of the neighborhood (urban, inner city – safety may be an issue versus rural areas where they may not be in close proximity)
- What factors lead to a child with food insecurities becoming obese?
  - Linked to types of food purchased above
  - Also if you are living in constant anxiety and not knowing when you will have food availability, may eat increased amounts when food is available
  - Reliance on school meals, which also tend to be processed, longer shelf life, etc.

**What If/Next Step Questions:**

- What if the 8 month old is admitted for a prolonged hospitalization? In a single parent household, how would they balance the care of both children?
  - Discuss the difficult decision parents must face when they have another child at home and one is hospitalized.
    - Single parent household: if no other family/friends available, may not be able to afford childcare for this; and even so would only be for a few hours; would not be able to stay at bedside 24 hours with hospitalized child.
    - Two parent household: if one parent were working, then other parent would also need to care for other children at home. Thus, still one parent may not be able to stay at bedside 24 hours with the hospitalized child.
    - Discuss remembering this before passing judgment on a parent for not being at bedside and the importance of engaging the parents by calling frequently with updates to maintain communication.
- Discuss transportation issues for the single parent in shopping for groceries and/or attending doctors’ visits.
  - For the larger chain or wholesale grocery stores that are at a distance, can be incredibly difficult for a parent to travel with children, especially if they have chronic medical needs.
  - Similarly, for doctors’ visits, think of the cost of transportation for parents before passing judgment on why they may have missed a doctor’s appointment.
- What if a medication was not covered by the child’s insurance?
  - Even if the cost of medication is $20-30, think about the overall budget and what would have to be eliminated to purchase that medication; perhaps a meal for the family. What if the medication cost $100-200? That would be the entire food budget for the family in comparison.

1. **Post-Exercise Survey:** At the conclusion of the activity, students completed an anonymous post-exercise survey regarding their own budgeting practices, the effectiveness of the exercise in meeting its objectives, and the ease of the virtual delivery (Appendix G).

**Table 1: Overall Budget – Common challenges/discussion points**

| **Housing** | **Cable Service/Internet** |
| --- | --- |
| You do not have renter’s insurance. If there were a flood, etc., the family would not be covered for any damage/losses. | Discussion whether cable is needed versus internet for tablets/devices (but then would not be able to use TV) |
| **Utilities** | **Health Insurance** |
| Water/electric/gas are included in the rent | Family is enrolled in Medicaid (parent and children). |
| **Telephone (house/cell)** | **Clothing** |
| Often discussion on whether a house/cell phone is needed given limited budget. Discuss need for phone for emergencies, especially with young children with chronic medical needs. | - Use of thrift/2^nd^ hand stores if available; potential donations at local centers. - Infant: Growing quickly and changes sizes every few months. |
| **Cleaning Products** | **Child Care** |
| - Difficulty traveling with two children eight blocks for laundry. - Need to include cost of detergent - Difficulty with young child still in diapers who has frequent accidents/soiling of clothes. | - If mother needs to go anywhere without the children (i.e. doctor’s visits, etc.), would need a babysitter/child care. - If one child is admitted, children often not allowed to visit (even pre-COVID) and thus would require childcare for other child. |
| **Personal Care/Hygiene Products** | **Medications** |
| If female parent, need to include feminine products.   - - Discussion about cost of pads/tampons vs menstrual cups, etc. | Medicaid pays for the family’s prescription medications (not include herbal remedies, vitamins, supplements) |
| **Recreation/Leisure** | **School Expenses** |
| - Movies - Ice cream outing - Birthday parties for classmates - Sports fees for older child | - Backpack - School supplies - School trips   Need to look out for backpack drives, etc. |
| **Transportation** | |
| - Zone refers to how far you want to ride on a particular bus/train; varies for each route. - The further away a specific destination is will require more zones to pass through and thus higher cost. - Transfer if you need to change buses/trains. | |
| **Food** | |
| You receive $535/month in SNAP benefits; they can be used for ANY foods except prepared hot foods. You cannot use them for non-food items (e.g. vitamins, diapers). See attached Food Spreadsheet for costs.  Resource: <https://www.fns.usda.gov/snap/eligible-food-items>  Types of foods and type of grocery store are common questions/challenges.   - Larger wholesale food stores sell items in bulk, which may utilize significant portion of budget at once; requires annual membership fee; potential for food to spoil if bought in bulk; cost of transportation (car share or taxi given size of items) and storage for these food items within a 1-bedroom apartment. - Local food stores within walking distance can be helpful as it eliminates travel cost/needs; however, products are often more expensive. - If parent not breastfeeding, added cost of formula   - Women, Infant and Children can provide formula if qualifies; discuss that this will provide a set amount each month; it is not an endless supply. - Examples include when parents run out of formula and then dilute formula to make it last longer; with higher water load; hypotonic fluids can cause hyponatremia and if severe, lead to seizures, etc. | |

**Table 2. Examples of stereotypes/biases**

| **Stereotype/Bias** | **Fact/Resource** |
| --- | --- |
| **Race/Ethnicity** | |
| Black individuals are the primary recipients through federal assistance programs. | 2019 % of Households Receiving Supplemental Nutrition Assistance Program (SNAP):  White, Not Hispanic or Latino: 45.6%  Black or African American: 26.8%  Hispanic or Latino (of any race): 21.2%  Asian 3.0%; American Indian and Alaska Native 1.6%  <https://data.census.gov/cedsci/table?q=s2201&tid=ACSST1Y2019.S2201&hidePreview=false> |
| Black neighborhoods are “crime-ridden, rundown, impoverished” while white neighborhoods are “safe, well-maintained, wealth”. | While compared to white Americans, black Americans are more likely to live in high-poverty neighborhoods, most black Americans do NOT live in high-poverty neighborhoods. "Poverty is not an essential quality of black neighborhoods. It's the cumulative effects of decades of federal and local policies that cut off black people’s access to resources, like home mortgages and well-resourced schools, that has led to the overrepresentation of black people among high-poverty neighborhoods." <https://news.ucsc.edu/2018/11/bonam-housing.html> |
| Hispanics/Latinos are all immigrants | 2017: 67% of the United States Hispanic population were born in the United States. Further, 79% of the entire United States Hispanic population are U.S. Citizens.  <https://www.pewresearch.org/hispanic/fact-sheet/latinos-in-the-u-s-fact-sheet/> |
| **Food Insecurities** | |
| Assumption that SNAP provide an endless supply of foods | SNAP/food stamps have a maximum amount based on # persons/family and is administered at the state level. This can only be used to purchase food and not prepared/hot items (no diapers, wipes, etc.). |
| Bias towards obese children/adults | Discuss types of foods purchased – high carbohydrates, processed, high sugar foods are cheaper, more filling, and have longer shelf lives. Fast food may be cheaper than purchasing same items at grocery store and cooking for yourself. |
| **Poverty** | |
| Individuals “choose” not to work | There are numerous potential barriers, including availability of jobs, childcare issues, lack of education or specific skill for jobs available, illness or disability for person or their children/relatives. |
| **Budgeting** | |
| Individuals living in poverty are wasteful of money. | Budgeting requires basic math skills and/or access to spreadsheets, etc. Reality is even with budgeting, when resources are limited, individuals must make tough decisions in prioritizing expenses for that month, which may mean falling behind on rent; parents not purchasing things for themselves, or limiting transportation costs (i.e. missing doctor’s appointments, etc.) |
| **Gender** | |
| The single parent is always the mother. | Assumption that the mother is the caregiver; or individual parent household, mother staying home is praised while father is looked down upon for staying home. |
| **Language/Immigration** | |
| If an individual does not understand/speak English, then they are not intelligent. | While this may seem obvious to us as health professionals, this can be an implicit bias. Remind students regarding importance of utilizing a certified interpreter (reflect on putting ourselves in the individual’s shoes and how it must feel to be in a hospital/healthcare setting and not receive communication in one’s primary language). |
| **Geography** | |
| Individuals living in Urban setting all have access to care. | While there may be more providers available within an urban setting, access to care includes insurance coverage, providers who accept the insurance, available hours, and quality of care. |

**References:**

1. DallaPiazza M, PadillaRegister M, Dwarakanath M, Obamedo E, Hill J, Soto-Greene ML. Exploring racism and health: an intensive interactive session for medical students. MedEdPORTAL. 2018;14:10783. [https://doi.org/10.15766/mep_2374- 8265.10783](https://doi.org/10.15766/mep_2374-%208265.10783)
2. U.S. Department of Agriculture Food and Nutrition Service. Supplemental Nutrition Assistance Program (SNAP). <https://www.fns.usda.gov/snap/recipient/eligibility> Accessed June 7, 2021.
3. Official Site of the State of New Jersey. Department of Human Services. NJ SNAP. <https://www.nj.gov/humanservices/njsnap/about/njsnap/> Accessed June 7, 2021.
4. U.S. Department of Agriculture Food and Nutrition Service. Special Supplemental Nutrition Program for Women, Infants, and Children (WIC). <https://www.fns.usda.gov/wic> Accessed June 7, 2021.
5. Social Security Administration. Supplemental Security Income (SSI) for Children. <https://www.ssa.gov/benefits/disability/apply-child.html> Accessed June 7, 2021.
6. HealthCare.gov. Immigrants: Coverage for lawfully present immigrants. <https://www.healthcare.gov/immigrants/lawfully-present-immigrants/> Accessed June 7, 2021.
7. U.S. Citizenship and Immigration Services. Green Card Eligibility Categories. <https://www.uscis.gov/green-card/green-card-eligibility-categories> Accessed June 7, 2021.
8. Ellis WR and Dietz WH. A new framework for addressing Adverse Childhood and Community Experiences: The Building Community Resilience Model. Academic Pediatrics, 2017: 17 (7) S86-S93. <https://doi.org/10.1016/j.acap.2016.12.011>
9. United States Census Bureau. Food Stamps/Supplemental Nutrition Assistance Program (SNAP). <https://data.census.gov/cedsci/table?q=s2201&tid=ACSST1Y2019.S2201&hidePreview=false> Accessed June 7, 2021.
10. McNulty J. Racial bias taints neighborhoods – and residents, research reveals. UC Santa Cruz Magazine. <https://news.ucsc.edu/2018/11/bonam-housing.html> Published November 28, 2018. Accessed June 7, 2021.
11. Noe-Bustamante L and Flores A. Facts on Latinos in the U.S. Pew Research Center. <https://www.pewresearch.org/hispanic/fact-sheet/latinos-in-the-u-s-fact-sheet/> Published September 16, 2019. Accessed June 7, 2021.
