# Supplementary material for: Down to the Last Dollar: Utilizing a Virtual Budgeting Exercise to Recognize Implicit Bias
Source: MedEdPORTAL. 2021 Dec 6;17:11199. doi: 10.15766/mep_2374-8265.11199 (PMC8645532; doi:10.15766/mep_2374-8265.11199)
Supplement: Supplementary file 1 — Social Determinants of Health Lecture.pptxCase Scenario with Group Reflection Exercise.docxBudgeting Templates - Common Food Prices.xlsxExample of Budget - Chain Grocery Store.xlsxExample of Budget - Wholesale Grocery Store.xlsxFacilitator Guide.docxSession Evaluation.docx [file mep_2374-8265.11199-s001.zip › G. Session Evaluation.docx]

**Appendix G**: Session Evaluation

# Do you currently utilize a personal budget when making regular purchases?


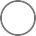
 Yes


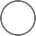
 No

# How many people do you currently shop for?


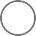
 0 (someone else does the majority of my regular shopping)


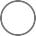
 1 (I shop for only myself, including online grocery shopping services such as Instacart, etc.)


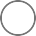
 2-3


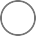
 4+

1. After completing the budgeting exercise, reflection, and group discussion, please rate the following.

Hardly at all

To a small degree

To a moderate degree

To a considerable degree

To a very high degree

The budgeting exercise allowed me to

recognize the effects of
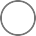

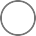

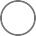

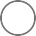

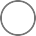
 poverty and food

insecurities on health.


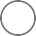

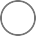

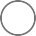

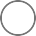

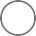
The budgeting exercise allowed me to recognize biases towards patients.


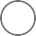

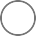

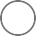

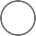

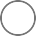
The reflection exercise allowed me to recognize biases towards patients.


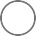

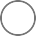

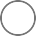

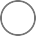

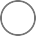
The group discussion enhanced my perspective about biases towards patients.

# After completing the budgeting exercise, reflection, and group discussion, please rate the following.

Strongly

Disagree Disagree Neutral Agree Strongly Agree

The activity was

conducted virtually
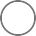

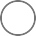

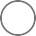

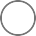

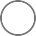
 without difficulty.


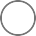

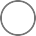

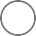

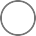

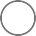
The time allotted was sufficient for the group to complete the budgeting exercise.


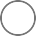

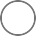

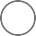

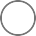

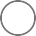
The content of this exercise relates to patients I have seen on the pediatric clerkship.

# What were the strengths of the curriculum?

1. What areas of the curriculum can be improved?
